# Natural variation of *HTH5* from wild rice, *Oryza rufipogon* Griff., is involved in conferring high‐temperature tolerance at the heading stage

**DOI:** 10.1111/pbi.13835

**Published:** 2022-05-25

**Authors:** Zhibin Cao, Huiwu Tang, Yaohui Cai, Bohong Zeng, Jialiang Zhao, Xiuying Tang, Ming Lu, Huimin Wang, Xuejing Zhu, Xiaofeng Wu, Linfeng Yuan, Jianlin Wan

**Affiliations:** ^1^ 205386 Rice National Engineering Research Center (Nanchang) Jiangxi Research and Development Center of Super Rice Jiangxi Academy of Agricultural Sciences Nanchang China; ^2^ 47894 College of Agriculture and Biology Zhongkai University of Agriculture and Engineering Guangzhou China

**Keywords:** rice, quantitative trait locus, near‐isogenic line, heat tolerance, map‐based cloning

## Abstract

Global warming is a major abiotic stress factor, which limit rice production. Exploiting the genetic basis of the natural variation in heat resistance at different reproductive stages among diverse exotic Oryza germplasms can help breeding heat‐resistant rice cultivars. Here, we identified a stable quantitative trait locus (QTL) for heat tolerance at the heading stage on chromosome 5 (*qHTH5*) in *O*. *rufipogon* Griff. The corresponding gene, *HTH5*, pertains to the pyridoxal phosphate‐binding protein PLPBP (formerly called PROSC) family, which is predicted to encode pyridoxal phosphate homeostasis protein (PLPHP) localized to the mitochondrion. Overexpression of *HTH5* increased the seed‐setting rate of rice plants under heat stress at the heading stage, whereas suppression of *HTH5* resulted in greater susceptibility to heat stress. Further investigation indicated that *HTH5* reduces reactive oxygen species accumulation at high temperatures by increasing the heat‐induced pyridoxal 5'‐phosphate (PLP) content. Moreover, we found that two SNPs located in the *HTH5* promoter region are involved with its expression level and associated with heat tolerance diversity. These findings suggest that the novel gene *HTH5* might have great potential value for heightening rice tolerance to heat stress to the on‐going threat of global warming.

## Introduction

Rice (*Oryza sativa* L.), is a major staple food crop providing energy for nearly half of total population in the world (Khush, 1997). It is commonly defined as a species originating in hot climate environment, including tropical and subtropical zones. (Yin *et al* .1996), but extreme high day and night temperatures during reproductive stages are major factors that limit rice production (Krishnan *et al*., 2011). When rice spikelets have undergone high temperatures (>35°C) for 1 h at the heading stage, high temperature seriously affects anther dehiscence and shedding pollen capacity, and cause spikelet sterility (Jagadish *et al*., 2007, 2010; Matsui *et al*., 1997, 2001; Matsui and Omasa, 2002; Satake and Yoshida, 1978; Shanmugavadivel *et al*., 2017). In recent years, significant efforts have been made to dissect the genetic mechanisms of heat tolerance, many QTLs/genes for heat resistance at different reproductive stages have been reported in rice by mutants or different genetic populations (Cao *et al*., 2020; Cheng *et al*., 2012; Chen *et al*., 2019; Kan *et al*., 2022; Li *et al*., 2015; Li *et al*., 2018; Seo *et al*., 2019; Vivitha *et al*., 2017; Wang *et al*., 2016; Xiao *et al*., 2011a; Xiao *et al*., 2011b; Xu *et al*., 2020; Ye *et al*., 2012; Ye *et al*., 2015a; Ye *et al*., 2015b; Zhao *et al*., 2016; Zhu *et al*., 2017), which has facilitated the breeding of heat‐tolerant rice varieties to deal with global warming. Among the known genes for heat tolerance, various regulatory mechanisms have been elucidated to minimize the harmful effects of heat stress, such as renaturation or elimination of denatured proteins, tRNA thiolation, maintaining the cell membrane structure, ROS scavenging, and retention of wax content in crop plants: *OsTT1* encodes the α2 subunit of the 26S proteasome, which is associated with the degradation of ubiquitinated proteins (Li *et al*., 2015); *SLG1* (*Slender Guy 1*) encodes a cytosolic tRNA2‐thiolation protein 2 involved in the tRNA (BR) thiolation pathway (Xu *et al*., 2020); *OsSIZ1* encodes a rice SUMO E3 ligase, which is involved in cell membrane integrity (Li *et al*., 2013 ); *OsANN1* encodes the calcium‐binding protein, which is involved in modulating the production of H_2_O_2_ (Qiao *et al*., 2015); and *TT2* (*THEROMOTOLERANCE 2*) encodes a Gγ subunit, which is associated with regulating metabolism of wax at high temperatures and which maintains greater heat retention (Kan *et al*., 2022). Pyridoxal phosphate homeostasis protein (PLPHP), plays a part in mitochondrial metabolism, and is crucial for the homeostatic regulation of pyridoxal 5'‐phosphate (PLP) in human cells and yeast (Johnstone *et al*., 2019). However, whether PLPHP participates in the homeostatic regulation of pyridoxal 5'‐phosphate (PLP) in rice against high‐temperature stress remains unclear.

In this study, we cloned and characterized a gene HTH5, which encodes a pyridoxal phosphate homeostasis protein (PLPHP). HTH5 also confirmed its positive regulatory role in response to heat stress through complementation, overexpression, and RNAi line tests at the heading stage; two single nucleotide polymorphism (SNP) variations upstream of *HTH5* are involved in its transcriptional expression level, resulting in diversity in heat tolerance by influencing ROS scavenging during high‐temperature stress. The favourable allele of *HTH5* provides the potential for rice heat tolerance improvement.

## Results

### Characterization of the heat‐tolerant near‐isogenic line (NIL), R05‐12‐01

The phenotypes of R05‐12‐01 and Sasanishiki showed no significant differences when planted in the Nanchang breeding station under normal open‐field‐ trial temperature conditions (Table S1). Significant differences between R05‐12‐01 and Sasanishiki were observed in pollen vitality, pollen germination rate on culture medium, mean seed‐setting rate per plant, and single plant yield after suffering heat stress at the heading stage. These results indicated that R05‐12‐01 closely resembles that of Sasanishiki in terms of major agronomic traits, but had increased heat tolerance during heading stage with a greater mean seed‐setting rate per plant and pollen vitality under heat stress conditions. Pearson correlation coefficient analysis of pollen vitality, pollen germination rate on culture medium, and mean seed‐setting rate per plant of R05‐12‐01 and Sasanishiki was conducted under high‐temperature stress during heading stage (Table S2). The mean seed‐setting rate per plant of Sasanishiki was significantly positively correlated with pollen vitality (*R^2^* = 0.893, *P *< 0.01) and the pollen germination rate in the culture medium (*R^2^* = 0.840, *P *< 0.01). The pollen germination rate in the culture medium of R05‐12‐01 was significantly positively correlated with pollen vitality (*R^2^* = 0.802, *P *< 0.01) and the pollen germination rate on the culture medium (*R^2^* = 0.849, *P *< 0.01). Therefore, the mean seed‐setting rate per plant is a reliable index that can be used to measure the trait of heat‐tolerance during the heading stage.

### Map‐Based Cloning of *qHTH5*

**Figure 1:**
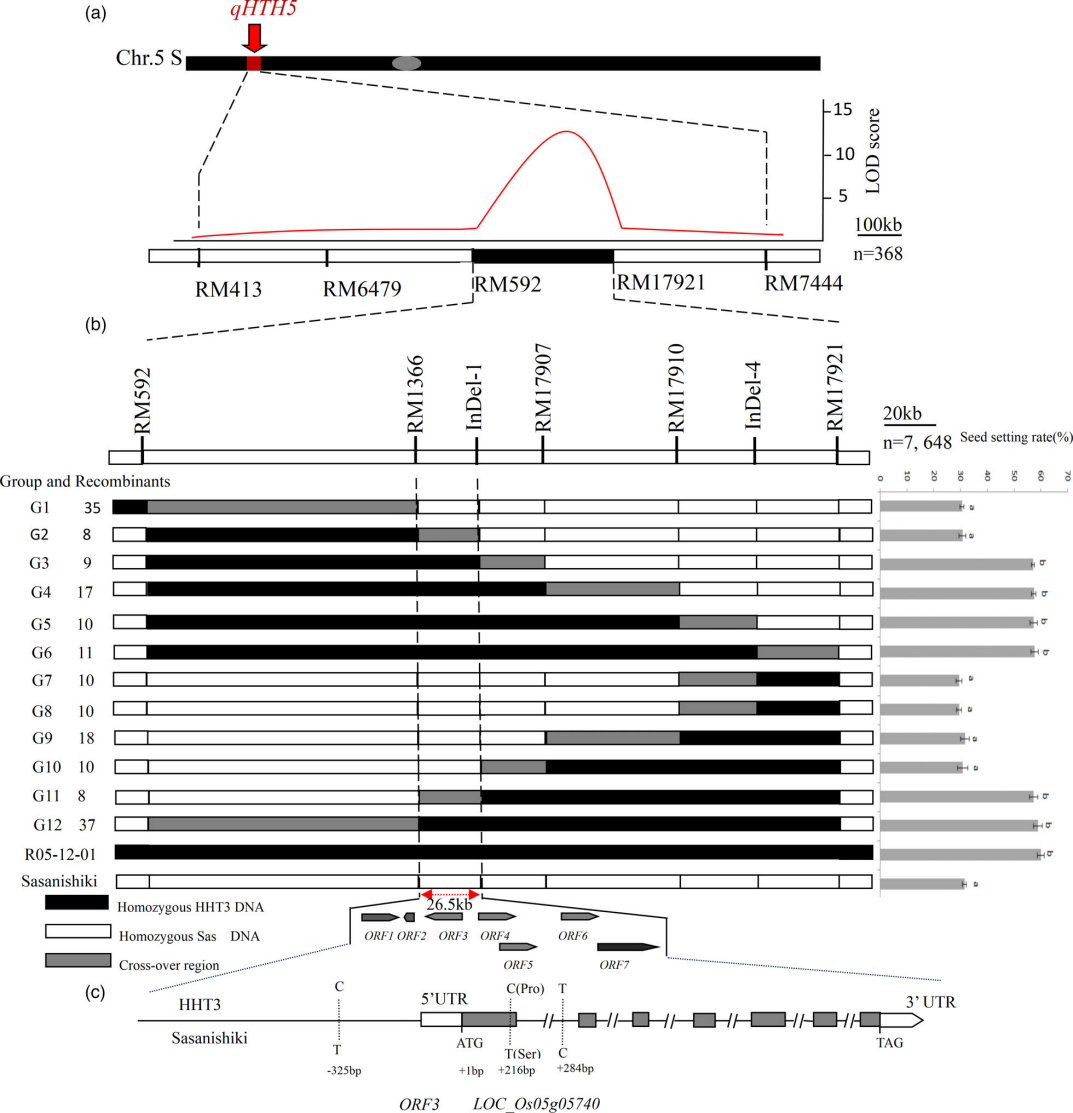
Map‐based cloning of *qHTH5*. Graphical genotype of introgression line on short arm of chromosome 5 used to generate BC_4_F_2_ population for segregation analysis. A high‐resolution physical map of *qHTH5* was developed for chromosome5 of BC_5_F_2_. The number of representatives is provided below the map, and the non‐significant difference between means was denoted by the presence of the same lowercase letter above the error bar (*P* > 0.05). *P*‐values were calculated with one‐way analysis of variance. Gene structure and natural variations of *HTH5*.

We carried out a QTL analysis in the BC_4_F_2_ population containing 368 individuals derived from a cross between Sasanishiki and R05‐12‐01 by composite interval mapping. We had obtained a significant peak between markers RM592 and RM17921, with an LOD value of 13.1; it explained up to 37.3% of the phenotypic variance (Figure 1a). The progeny test of the population containing 64 BC_4_F_3_ families showed that AA homozygous for HHT3, heterozygous Aa families, and aa homozygous for Sasanishiki were 13, 37, and 14, respectively. Furthermore, it was showed that the counts of the three categories were fit with an expected single‐locus Mendelian segregation ration (1:2:1 by progeny test, χ^2^ = 1.16 < χ^2^_0.05, 2_ = 5.99) (Table S3). It indicated that *qHTH5* was a stable locus, and the QTL’s position was consistent with our published mapping result (Cao *et al*., 2015).

To further isolate *qHTH5*, high‐resolution mapping was performed by screening a larger BC_5_F_2_ population containing 7, 648 individuals (Figure 1b). Among the 7, 648 individuals, 183 recombinants between RM592 and RM17921 were detected for further analysis. Twelve types of recombinants (G1‐G12) were grouped based on the genotypic groups of markers in the region between RM592 and RM17921. For fine mapping *qHTH5*, the mean seed‐setting rate of the main panicles of 183 recombinants for heat response from each group were compared to R05‐12‐01 and Sasanishiki. The mean seed‐setting rate of main panicles in groups G1and G2 were significantly lower (*P *< 0.01) than that of R05‐12‐01. For G3, the mean seed‐setting rate of the main panicles of recombinants was significantly higher (*P* < 0.01) than that of Sasanishiki. Therefore, *qHTH5* was located to the downstream side of marker RM1366. Similarly, groups G10 and G11 delimited *qHTH5* upstream of the insertion and deletion (InDel) marker InDel‐1. Combining the dataset of comparing the mean seed‐setting rate of main panicles of the other recombinant groups, we finally delimited *qHTH5* to a 26.5‐kb region between markers RM1366 and InDel‐1. The Progeny test of homozygous recombinants (BC_5_F_3_) also delimited *qHTH5* to the same region according to the above method (Figure S2).

In this genomic region, there were seven predicted ORFs according to the Nipponbare genome sequence from Rice Genome Annotation Project(RGAP) database (http://rice.plantbiology.msu.edu.cn/; Table S4). qRT‐PCR was used to analyse the expression levels of the seven genes encoding proteins in R05‐12‐01 and Sasanishiki suffering heat stress for different time periods under the controlled conditions (Figure S3a–g). It was found that only one open reading frame (ORF), *LOC_Os05g05740*, which encodes a putative proline synthetase co‐transcribed bacterial homologue protein (PROSC), showed significantly higher expression levels at 1–16 h in the panicle tissue of R05‐12‐01 compared to Sasanishiki induced by heat stress at the heading stage (Figure S3c). Sequence analysis showed that the coding sequence of *HTH5 (LOC_Os05g05740)* consisted of seven exons with a cDNA sequence of 732 bp. One SNP was found in the promoter region and 3′‐untranslated region, and two SNPs were found in the genomic region of *LOC_Os05g05740* between HHT3 and Sasanishiki, and the SNP in exon1 resulted in an amino acid change (Figure 1c). Therefore, we speculated that *LOC_Os05g05740* as an candidate gene might be responsible for *qHTH5*, hereafter referred to as *HTH5*.

BLASTP analysis of the NCBI database using our sequencing results showed that *HTH5* encoded a pyridoxal phosphate homeostasis protein, which contains a PLP‐binding barrel domain and exhibits high structural similarity with the N‐terminus of bacterial alanine racemase and eukaryotic ornithine decarboxylase (Ito *et al*., 2013; Prunetti *et al*., 2016). The sequence alignment analysis showed that *HTH5* was a highly conserved pyridoxal phosphate homeostasis protein in higher plants, and even in eukaryotes (Figures S4 and S5). Among these orthologous proteins, *HTH5* orthologues shared high similarity (PI > 80%) with those in monocots and dicots, whose *HTH5* orthologue protein similarity ranged from 86 to 98% and 84 to 90%, respectively. The *HTH5* orthologues of human, mouse, yeast, and roundworm possessed protein similarities of 64%, 67%, 74%, and 75%, respectively (Figure S5). According to the sequence alignment of the NCBI database, there is only one gene encoding PLPHP in rice. These results demonstrated that there is only one copy of *HTH5* in rice, and that *HTH5* is highly conserved in plants, suggesting that it may have a critical function in the plant kingdom.

### *HTH5* is a positive regulator of heat tolerance

**Figure 2:**
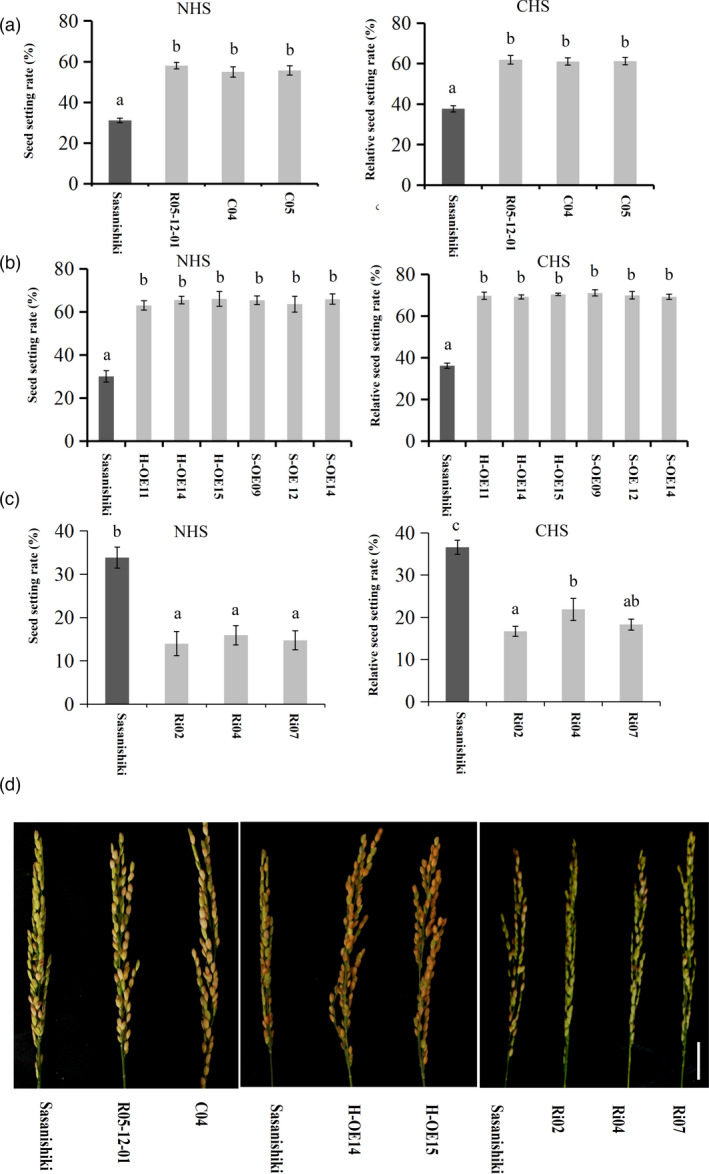
Functional characterization of *HTH5* at the heading stage. Statistical results for the mean seed‐setting rate of the main panicles of Sasanishiki, R05‐12‐01, and complementation lines under natural heat stress (NHS) and controlled heat stress (CHS). Data represent mean ± SD (*n *= 10). Statistical results for the mean seed‐setting rate of the main panicles of Sasanishiki and overexpression plants by heat treatment. Data represent mean ± SD (*n *= 10). (c) Statistical results for the mean seed‐setting rate of main panicles of Sasanishiki and RNAi plants by heat treatment. Data represent mean ± SD (*n *= 10). (d) Phenotype of panicles from Sasanishiki, complementation lines, overexpression lines, and RNAi lines under heat stress. Data represent mean ± SD (*n *= 10). Scale Bar, 2cm. Significance was determined by one‐way ANOVA, comparisons of means were conducted using Tukey’s test (*P* < 0.05). The non‐significant difference between the means was denoted by same lowercase letters above the error bar (*P* > 0.05, Student’s *t* test was performed for calculating *P*‐values ).

To confirm our prediction of the gene *HTH5*, which is responsible for *qHTH5*, a complementary test construct was introduced into Sasanishiki. Compared to CK, complementary test transgene‐positive plants, C04 and C05, in the T_1_ progeny for *HTH5^HHT3^*, showed a significantly increased expression level in anthers (Figure S6a). Phenotypic analysis of the two transgenic lines C04 and C05 in the T_2_ progeny showed significantly higher heat tolerance than Sasanishiki under controlled and normal open field heat stress conditions at the heading stage (Figure 2a, d). Therefore, we conclude that *HTH5* is a functional gene in *qHTH5*. Second, two overexpression constructs containing the *HTH5* allele from HHT3(H‐OE) or Sasanishiki (S‐OE) were separately transformed into Sasanishiki. Compared to CK, overexpression transgene‐positive plants, H‐OE11, H‐OE14, H‐OE15, S‐OE09, S‐OE12, and S‐OE14 lines, in the T_1_ progeny for *HTH5*, showed significantly increased expression levels in anthers (Figure S6b). Phenotypic analysis showed that the H‐OE11, H‐OE14, and H‐OE15 lines in T_2_ progeny showed higher heat tolerance compared to Sasanishiki under controlled and normal field trial heat stress conditions, and S‐OE09, S‐OE12 and S‐OE14 lines also possessed enhanced heat tolerance at the heading stage with significantly higher seed‐setting rate (Figure 2b, d; Figure S6c), and they were no less than H‐OE lines according to statistical results for the mean seed‐setting rate of main panicles (Figure 2b). To silence the expression of *qHTH5*, we have developed an RNA interference (RNAi) construct and transformed into Sasanishiki. Compared to CK, transgene‐positive plants Ri02, Ri04, and Ri05 lines in the T_1_ progeny for *HTH5* showed significantly decreased expression levels in anthers (Figure S6d). Down regulation of *HTH5* of Sasanishiki caused a significant reduction in the seed‐setting rate of the Ri02, Ri04, and Ri05 lines in the T_2_ progeny compared to the wild type under normal field trial heat stress and controlled heat stress conditions (Figure 2c, d). Meanwhile, it was found that there were no significant differences between the Sasanishiki and overexpression transgenic plants in yield‐related agronomic traits, including grain length, grain width, grain thickness, and number of effective panicles per plant, number of total grains per panicle, and 1000‐grain weight (Figure S7a–e). We also found that *HTH5^HHT3^* overexpression improved rice grain yield under heat stress (Figure S7f–h). These results showed that *HTH5* is responsible for *qHTH5*.

### Higher expression level of *HTH5* enhancing heat tolerance

**Figure 3:**
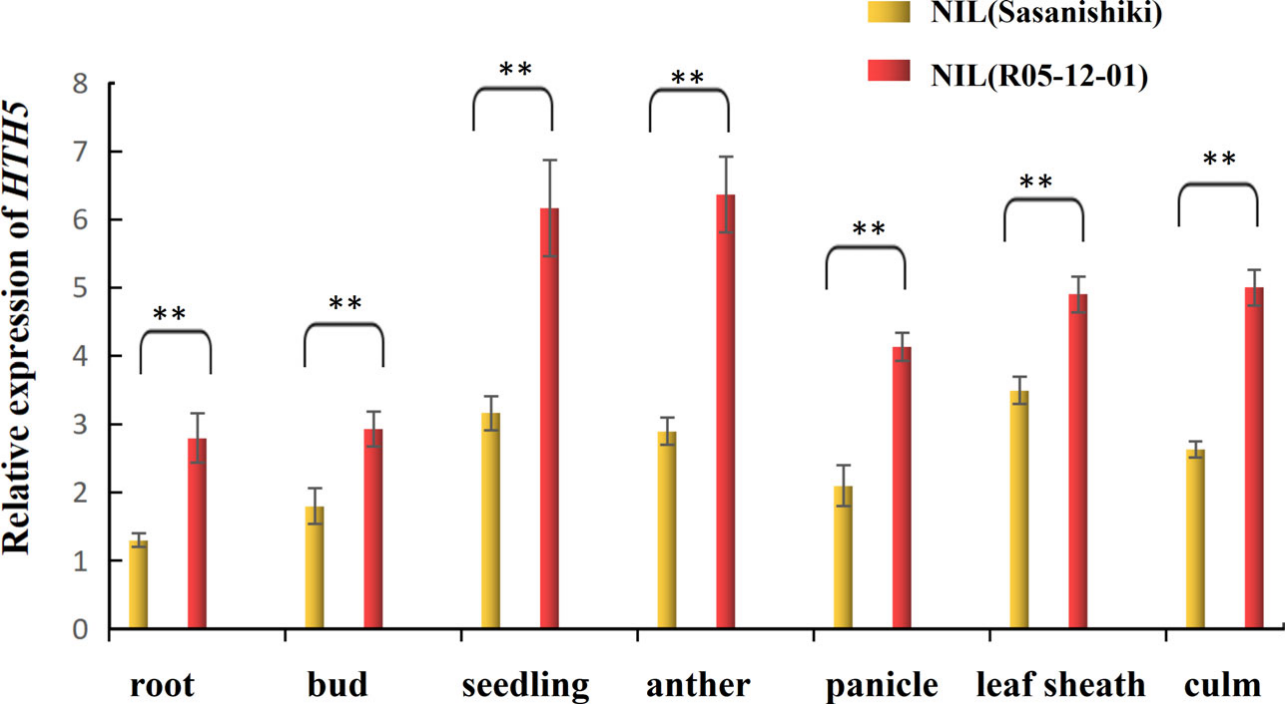
Expression patterns of *HTH5*. qRT‐PCR was performed to analysis the expression levels of *HTH5* in different tissues of R05‐12‐01 and Sasanishiki. Data represent mean ± SD (*n *= 3). Student’s *t* test ** indicates significant differences at the 0.01 level.

To illustrate how *HTH5* regulates heat tolerance, qRT‐PCR was used to examine the expression patterns of *HTH5* in all rice tissues of NIL‐Sasanishiki and NIL‐ R05‐12‐01. The analysis of qRT‐PCR data showed that the expression level of *LOC Os05g05740* was differentially expressed in NIL‐R05‐12‐01 and NIL‐Sasanishiki, and significantly higher expression level was induced by heat stress (Figure S3c). Moreover, it was widely expressed in buds, roots, anthers, panicles, leaf sheaths, and culms, especially in seedling leaves and anthers, and had significantly higher relative transcript expression levels in NIL‐R05‐12‐01 than in the corresponding NIL‐ Sasanishiki during the developmental process (Figure 3).

GUS histochemical staining analysis of pHTH5^HHT3^::GUS transgenic plants also revealed that *LOC_Os05g05740* was expressed in buds, roots, young leaves, spikelet hulls at the booting stage, elongation internode at the heading stage, flag leaf at the heading stage, mature anthers before dehiscence and leaf sheath (Figure S8a–i). The high expression in the young leaves and anthers was consistent with qRT‐PCR results, and higher GUS activity in the leaf sheath was also observed in response to heat stress (Figure S8i). Expression analysis also demonstrated that the promoter of the *HTH5* allele from HHT3 possessed greater GUS activity than that in Sasanishiki under heat stress during heading stage (Figure S9). Combined with the overexpression analysis of coding regions of *HTH5^HHT3^* and *HTH5^Sas^* (Figure 2b), we reasoned that the different expression levels of *HTH5* implied that sequence variation in the promoter region might affect the expression level of *HTH5*, thus leading to diverse heat tolerance.

### *HTH5* encodes a mitochondrion‐targeted protein PLPHP

**Figure 4:**
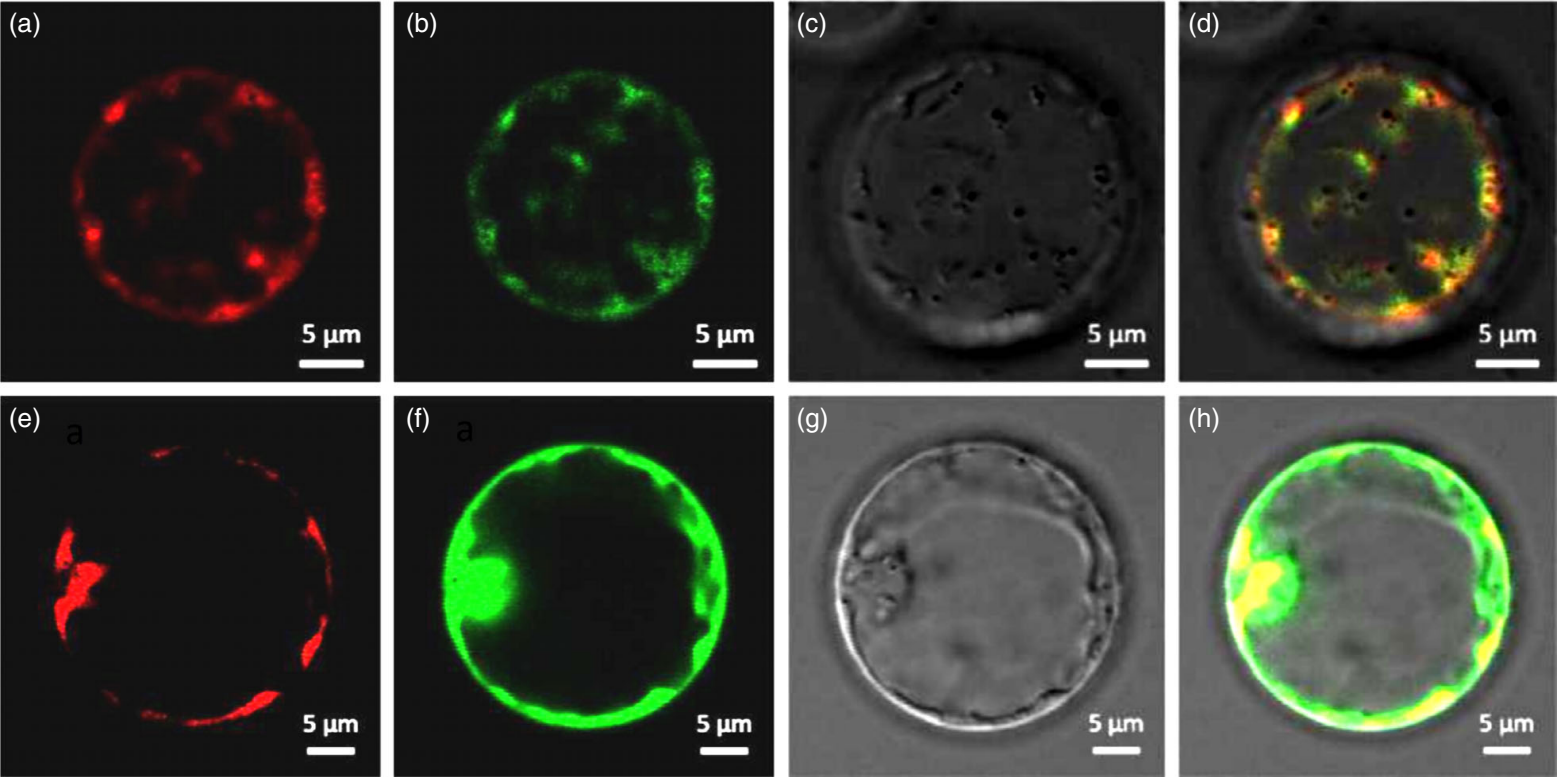
Subcellular localization of *HTH5*. (a, d) Mito Tracker red fluorescence. (b, f) GFP fluorescence. (c, g) Bright image. (d, h) Merged image. (a‐d) P35S::HTH5‐GFP. (e‐h) P35S::GFP.

It is reported that PLPHP is targeted to the mitochondria in yeast and human cells and can play a role in mitochondrial energy metabolism (Johnstone *et al*., 2019). To explore the subcellular localization of HTH5, full‐length HTH5 was fused with GFP in the pMDC83 vector. We transiently expressed the fusion protein in Arabidopsis protoplasts by bombarding, and detected the fluorescent signal using confocal laser microscopy (Figure 4). GFP signals were colocalized in small dots with the red fluorescence of MitoTracker pBIN20‐MT‐RK (Nelson *et al*., 2007). This result showed that HTH5 encodes a mitochondrion‐targeted protein PLPHP.

### The effect of PLP content on the heat response

**Figure 5:**
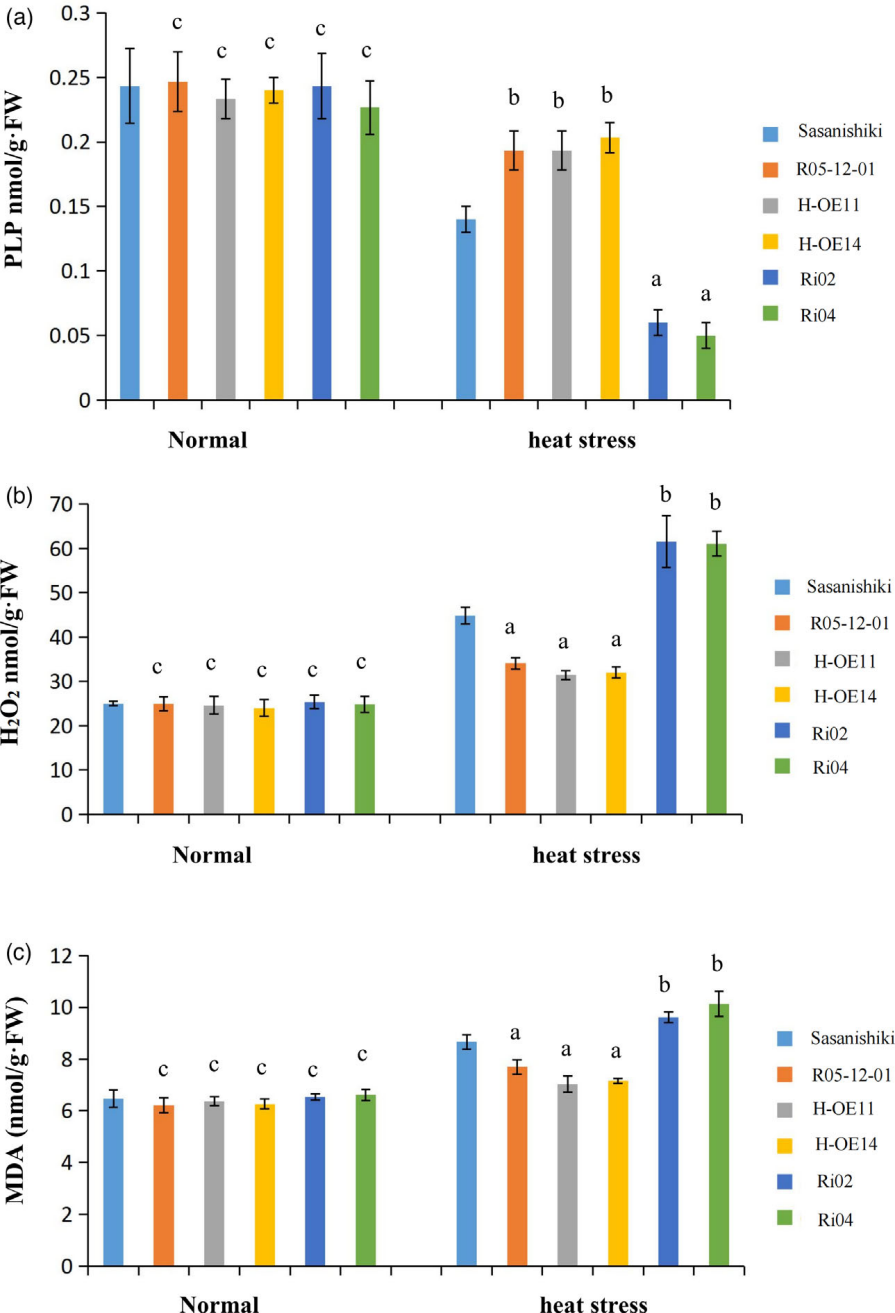
The heat response in rice transgenic lines and control line after heat stress. (a to c) Relative PLP content (a), H_2_O_2_ content (b), and MDA content (c) present in pollen grains from Sasanishiki, R05‐12‐01, HTH5‐OE, and RNAi transgenic lines at the heading stage under normal conditions (left, 28 °C for 12 h) and heat stress treatment (right, 38 °C for 12 h). Data are mean ± SD (*n *= 3). FW, fresh weight. Student’s *t* test. The letter a, b, and c above the bar indicates significantly lower than control at *P* < 0.01, higher than control at *P* < 0.01, and difference at *P* ≥ 0.05, respectively.

Several recent studies have shown that PLPBP may act a role as pyridoxal 5‐phosphate (PLP)‐binding protein (Ito *et al*. 2019; Prunetti *et al*.^2016^). To explore the potential mechanism of action of *HTH5*, which encodes a PLP‐binding protein and influences heat tolerance, we detected PLP content in pollen grains from Sasanishiki (NIL), R05‐12‐01, H‐OE11, H‐OE14, Ri02, and Ri04 lines under normal and heat stress conditions as depicted in Figure 5a. Under normal temperature conditions, the PLP content was nearly the same in all lines, and no significant difference was found to compared with the control group. Meanwhile, the statistical results showed that the heat stress condition caused a decrease in PLP content in all lines, but the R05‐12‐01, H‐OE11, and H‐OE14 lines showed significantly higher PLP content compared with that of Sasanishiki, and the RNAi lines (Ri02 and Ri04) also showed a significant reduction in PLP content than that of Sasanishiki. These results indicate that heat‐induced expression of *HTH5* boosts free PLP production. The statistical results of the seed‐setting rate from R05‐12‐01 and overexpression transgene‐positive lines (H‐OE11 and H‐OE14) under heat stress conditions at the heading stage, demonstrated that heat tolerance was superior to that of Sasanishiki. Therefore, these data clearly demonstrate that the relatively lower levels of PLP in grain pollen can reduce heat tolerance at the heading stage.

### ROS levels are regulated by PLP during high temperature stress

**Figure 6:**
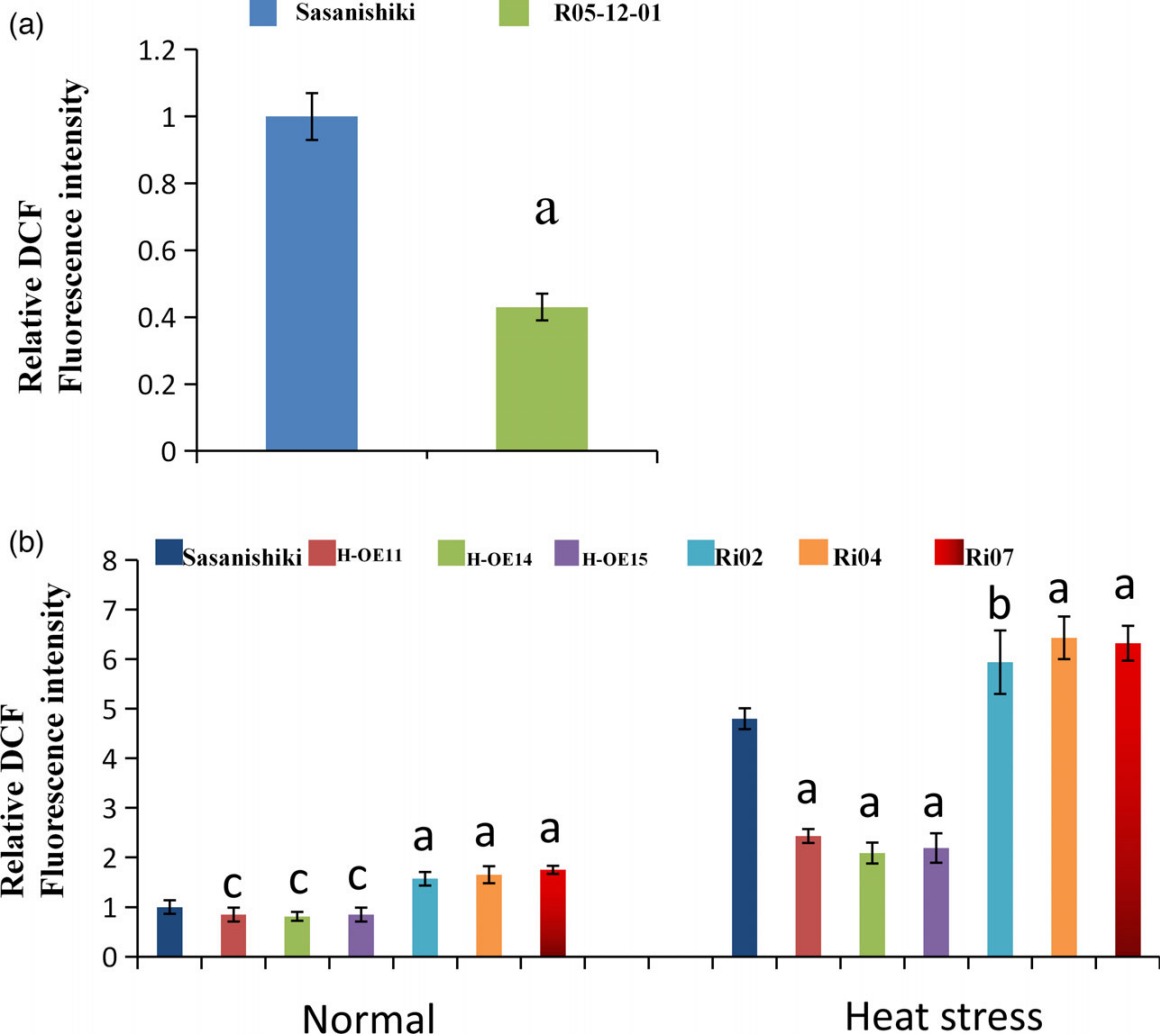
*HTH5* is involved in the ROS scavenging under heat stress treatment. Quantification of DCF fluorescence in pollen grains stained with the generic ROS sensor CM‐H_2_DCFDA of Sasanishiki and R05‐12‐01under heat stress treatment (38 °C /12 h). (b) Quantification of DCF fluorescence in pollen grains stained with the generic ROS sensor CM‐H_2_DCFDA from Sasanishiki, HTH5‐OE, and RNAi lines under normal temperature and heat stress treatments. (a and b) Data are presented as the mean ± SEM. Student’s *t* test. The letter a, b, or c bove the bar indicates significantly lower than control at *P* < 0.01, higher than control at *P* < 0.01, or difference at *P* ≥ 0.05, respectively. The normal condition and heat stress treatment was 28 °C and 38 °C, respectively for 12 h before anthesis.

To explore whether PLP contributes to heat tolerance by mediating antioxidant activity, the distribution of ROS in the pollen grains were examined by ROS sensors. The     general ROS sensor 5‐(and 6)‐chroromethyl‐2’, 7’‐dichlorodihydro‐fluorescein diacetate (CM‐H_2_DCFDA), which is converted to the highly fluorescent 2’, 7’‐dichlorodihydrofluorescein (DCF) upon oxidation by ROS, was used to visualised and quantified the total ROS. The DCF signal for the entire grain was quantified by LSCM. DCF fluorescence intensity was 2.3‐fold higher in Sasanishiki pollen grains than in R05‐12‐01 under the heat stress treatment (Figure 6a). To test whether *HTH5* increases rice heat tolerance through regulating ROS homeostasis, Sasanishiki (CK), HTH5‐OE, and RNAi plants were processed under normal and heat stress conditions, and quantitatively detected ROS accumulation followed by the general ROS sensor CM‐H_2_DCFDA. As shown in Figure 6b, the production of ROS in HTH5‐OE pollen grains was significantly lower than that in Sasanishiki, while the accumulation of ROS in HTH5‐RNAi plants was significantly greater than that in Sasanishiki under heat stress conditions. These results show that the overexpression of *HTH5* can significantly level down the accumulation of ROS arising from heat stress. Furthermore, we assessed the H_2_O_2_ content of pollen grains at the heading stage in Sasanishiki, R05‐12‐01, HTH5‐OE (H‐OE11 and H‐OE14), and RNAi (Ri02 and Ri04) plants after normal and high temperature treatments. As shown in Figure 5b, under normal temperature conditions, the RNAi transgene‐positive plants showed that the H_2_O_2_ content in Ri02 and Ri04 plants was 23.96 and 25.36 nmol/g·FW, respectively, and no significant difference was found in comparison with Sasanishiki (24.99 nmol/g·FW). Furthermore, the assessment results of R05‐12‐01 and overexpression transgene‐positive plants in T_3_ progeny for *HTH5* showed that the H_2_O_2_ content in R05‐12‐01, H‐OE11, and H‐OE14 were 24.93, 24.63, and 23.97 nmol/g·FW, respectively, and no significant difference was found in comparison with Sasanishiki (24.99 nmol/g·FW). The test results under high temperature treatment indicated higher H_2_O_2_ content in RNAi transgene‐positive plants in T_3_ progeny than in Sasanishiki plants after heat stress, and the H_2_O_2_ content in Ri02 and Ri04 was found to be 61.53 and 61.12 nmol/g·FW, respectively, which was significantly greater than that of Sasanishiki plants (44.83 nmol/g·FW). The H_2_O_2_ content in R05‐12‐01 plants was significantly lower than that in Sasanishiki plants after treatment with heat stress (34.10 vs. 44.83 nmol/g·FW). Similarly, the H_2_O_2_ content in overexpression transgene‐positive H‐OE11 and H‐OE14 was 31.43 and 32.03 nmol/g·FW, respectively, which was significantly lower than that found in Sasanishiki plants after treatment with heat stress (44.83 nmol/g·FW). Further, we studied whether lipid peroxidation of pollen grains under heat stress treatment was altered in Sasanishiki, R05‐12‐01, HTH5‐OE and RNAi plants. During the heading stage, we used malondialdehyde (MDA) content as an index of lipid peroxidation level, and Sasanishiki, R05‐12‐01, HTH5‐OE, and RNAi plants were also measured (Figure 5c). These results showed that MDA content in R05‐12‐01, HTH5‐OE, and RNAi plants was not significantly different from that in Sasanishiki plants (6.46 nmol/g·FW) under normal temperature condition. The MDA content in R05‐12‐01, H‐OE11, H‐OE14, Ri02, and Ri04, was found to be 6.21, 6.24, 6.26, 6.53, and 6.49 nmol/g·FW, respectively. The measured results under heat stress treatment showed significantly increase in MDA content in RNAi plants, and the MDA content in Ri02 and Ri04 was found to be 9.49 and 9.98 nmol/g·FW, respectively, which was significantly greater than that in Sasanishiki plants (8.66 nmol/g·FW). The MDA content in R05‐12‐01 and HTH5‐OE plants was significantly lower than that in Sasanishiki plants. The MDA content in R05‐12‐01, H‐OE11, and H‐OE14 was found to be 7.84, 6.74, and 7.26 nmol/g·FW, respectively. These results demonstrate that the heat sensitivity of the tested lines was brought about owing to oxidative stress.

### SNP variation in the promoter is associated with *HTH5* transcriptional level and heat tolerance

**Figure 7:**
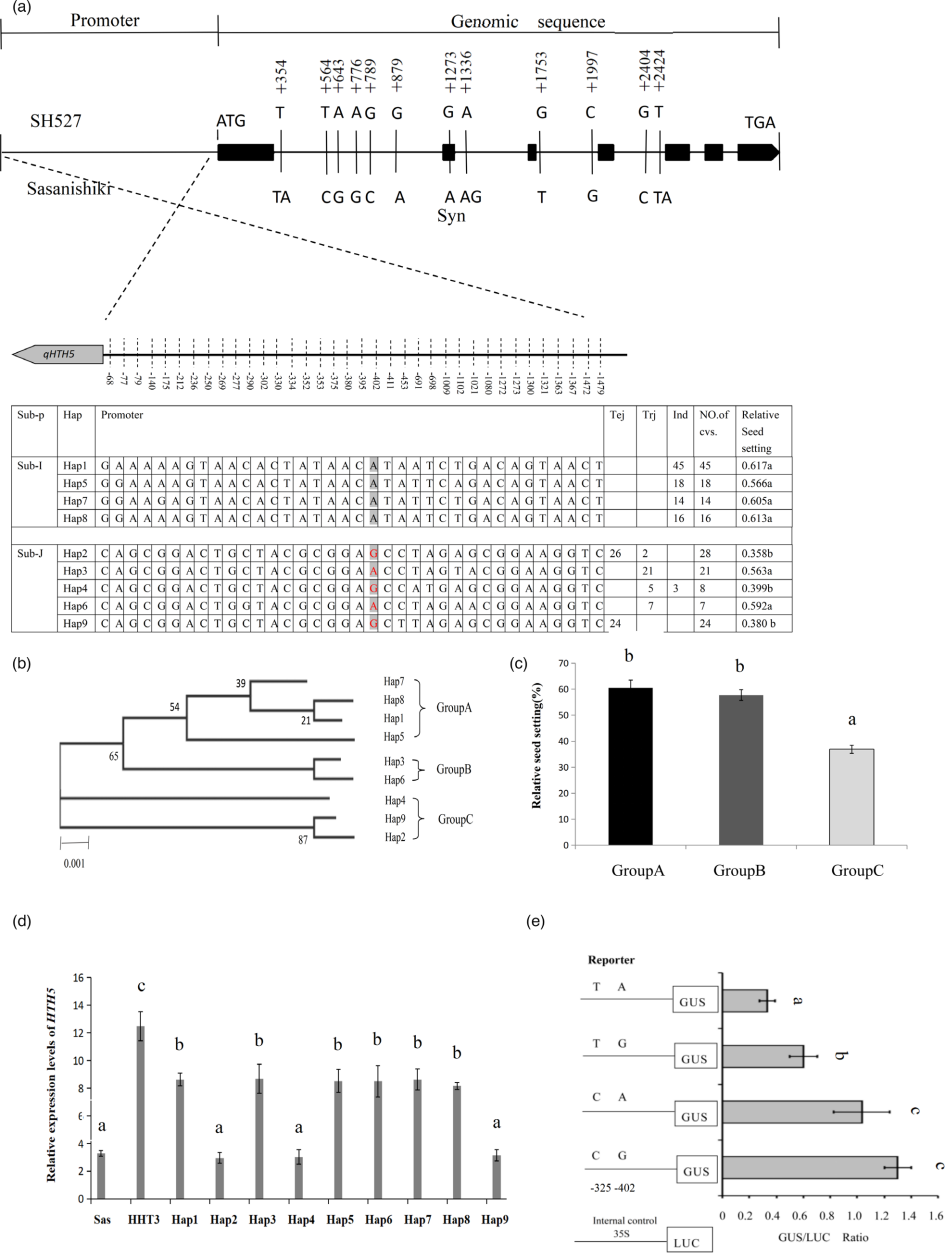
Haplotype analysis of *HTH5*. Haplotype analysis of the *qHTH5* region in 181 accessions. All the haplotypes were present in indica population (Ind), japonica sub‐population (Sub‐J), indica sub‐population (Sub‐I), temperate japonica population (Tej), and tropical japonica population (Trj). The data represent mean ± SD (*n *= 5). (b) According to the phylogenetic tree analysis, the nine haplotypes were classified into groups A (Hap1, Hap5, Hap7 and Hap8), B (Hap3 and Hap6), and C (Hap2, Hap4 and Hap9). The average number of substitutions per site was indicated by the scale bar. (c) Relative seed‐setting rate of the A, B and C groups. Data represent mean ± SD. (d) The relative expression level of Sasanishiki, NIL (R05‐12‐01), and nine haplotypes; three replicates were used for analysing each cultivar or line. (e) Promoter activity assay in Arabidopsis transient protoplasts. Left, two SNPs in the promoter region were site‐directed mutated for construction of vector. Right, relative GUS/LUC values. Significance was determined using ANOVA, comparisons of means were conducted using Tukey’s test (*P* < 0.05). The non‐significant difference between the means was denoted with same lowercase letters above the error bar (*P* > 0.05, *P*‐values were calculated using Student’s *t* test).

In our previous study, *qHTH5^HHT3^* under the *indica‐type* genetic background of Shuhui527 (SH527) was associated with an approximately 12% improvement in seed‐setting rate under heat stress at the heading stage (Cao *et al*., 2015). qHTH5HHT3 under japonica‐type genetic background of Sasanishiki can increase the seed‐setting rate by ~30% under the heat stress at the heading stage. To explore the differences in the genetic effects of the HHT3 allele and Sasanishiki allele, we sequenced and compared the genomic region of *qHTH5* from Sasanishiki and SH527, and we found no missense mutations between the two cultivars in the coding regions and no nucleotide difference in the 3’UTR of *qHTH5* (Figure 7a). We explored the relationship between heat tolerance and mutations in the promoter region of *HTH5* in diverse germplasms. First, we evaluated the heat tolerance at the heading stage of 181 accessions from different countries, including 50 temperate japonica, 35 tropical japonica, and 96 indica accessions (Figure 7a; Table S5). As expected, indica cultivars showed greater heat tolerance than japonica cultivars. Based on the nucleotide differences in SH527 and Sasanishiki, we divided the SNPs of the 181 accessions into nine haplotypes. It was found that Hap1(Hap‐SH527), Hap5, Hap7 and Hap8 only in sub‐I, and Hap2(Hap‐Sas), Hap3, Hap6, and Hap9 only in the sub‐J, and Hap4 both in sub‐J and sub‐I subpopulations (Figure 7a). Furthermore, the nine haplotypes could divide into three groups according to phylogenetic analysis; Hap1, Hap5, Hap7, and Hap8 in Group A, Hap3 and Hap6 in Group B, and Hap2, Hap4, and Hap9 in Group C (Figure 7b). Accessions in Groups A and B exhibited significantly greater heat tolerance at the heading stage than in Group C (Figure 7c). To test the effect of these SNP mutations on promoter, we explored transcriptional diversity of *HTH5* in these nine haplotypes and NILs (Sasnishiki and HHT3) in panicle tissue at heading stage under heat stress (38°C/12 h). The results indicated that the cultivars possessing Hap1 to Hap9 all tended to show significantly lower expression levels than NIL (HHT3); the cultivars possessing Hap2, Hap4, and Hap9 also tended to show significantly lower expression levels than other haplotypes (Figure 7d). Furthermore, we calculated the correlation between the relative expression levels of *HTH5* in different haplotypes and the seed‐setting rate under heat stress at the heading stage. We found that the relative seed‐setting rates of different haplotypes under controlled heat stress conditions at the heading stage had a significant positive correlation (r = 0.977) with the relative expression level of *HTH5*. To reduce the interference from population structure, we conducted an association test according to the SNPs of *HTH5* in 89 rice accessions from China, and also detected a strong signal at SNP‐402bp (Fisher’s exact test <0.01) in the promoter region (Figure 7a; Table S5 ).

These results suggest that the nucleotide mutation −402SNP in the promoter region is associated with heat tolerance diversity at the heading stage. Thus, the haplotypes of *HTH5* may divide into two types according to the SNP variation; the low expression type (Hap2‐Sas, Hap4, and Hap9), and the high expression type (Hap1‐SH527, Hap3, Hap5, Hap6, Hap7, and Hap8). However, the −325SNP variation in the promoter region of *HTH5^HHT3^* was not found in the cultivars tested; thus, we propose that *HTH5^HHT3^* is specific to the HHT3 allele, which elevates *HTH5* expression.

To determine whether these two SNPs (−402SNP and −325SNP) in the promoter region affected the expression of *HTH5*, we developed a series of pHTH5::GUS fusion constructs by inserting mutant promoter fragments and introducing them into *Arabidopsis* protoplasts for transient expression. The transient gene expression analysis showed that the −402SNP variation (constructs with A to G) in a mutant Sasanishiki promoter fragment exhibited significantly higher relative expression activity than that of the Sasanishiki promoter(Figure 7e). Similarly, transient expression assays of the site‐directed mutated promoter fragments were performed to test the effect of the −325SNP variation (constructs with T to C) in regulation to *HTH5* expression. Compared the expression activity of the promoter, the −325SNP variation (constructs with T to C) in a mutant Sasanishiki promoter fragment significantly increased its relative expression activity. We found that −325SNP variation carried cis‐element (TC‐rich repeats cis‐acting element involved in defense and stress responsiveness) changes by PLACE (Plant cis‐regulatory DNA elements) analysis, suggesting that these SNPs in the *HTH5* promoter were key for its transcript expression level in rice, and the −325SNP variation might account for the different heat tolerance effects of *HTH5^HHT3^* in the background of Shuhui527 and Sasanishiki.

To further confirm the transcriptional activities of the parents and the nine types (Hap1‐Hap9) of promoters under heat stress, we conducted transient expression assays in *Arabidopsis* protoplasts. Compared with the Sas‐type, the Hap2, Hap4, and Hap9 promoters, the HHT3‐type, and the Hap1, Hap3, Hap5, Hap6, Hap7 and Hap8 promoters were highly induced by heat stress. The HHT3‐type promoter was induced by 4.8‐fold in terms of relative expression activity compared with the normal control. The average relative expression activity of the Hap1, Hap3, Hap5, Hap6, Hap7 and Hap8 promoters was 2.9‐fold higher than that of the control (Figure S10a). Furthermore, it was found that the heat tolerance of different promoters (Hap1‐Hap9) exhibited a high positive correlation (*R* ^2^ = 0.9782) with relative expression activity driven by *HTH5* promoters (Figure S10b). These results indicate that the differential expression levels detected among the heat‐tolerant rice cultivars resulted from distinct responses from their promoters.

## Discussion

### *HTH5*, a new heat tolerance gene at the heading stage

Heat‐induced spikelet sterility often has a severe impact on rice yield worldwide during the heading stage (Hirabayashi *et al*. 2015). To date, more than 60 QTLs for heat tolerance traits in rice have been identified, but only two major QTLs for heat tolerance at the seedling stage (*OgTT1* and *OsHTAS*) have been cloned from genetic mapping populations using a classic map‐based approach. Even though great efforts have been made, the mechanisms that establish the final heat tolerance at the reproductive stage remain poorly understood. In this study, we used a map‐based cloning approach for heat tolerance at the heading stage based on high‐resolution mapping from a large NIL‐F_2_ segregation population and identified a major QTL, *qHTH5*, for heat tolerance at the heading stage from HHT3. In this study, we considered the relative seed‐setting rate as an index associated with heat tolerance at the heading stage under artificially controlled conditions, and developed NILs for *HTH5* in *indica‐type* and *japonica‐type* genetic backgrounds, aiming for genetic improvement of heat tolerance. Subsequently, the stably inherited QTL, *qHTH5*, was delimited to a 26.5kb DNA fragment in the target region. Thus, a new heat‐tolerance gene at the heading stage, *HTH5*, encoding a pyridoxal phosphate homeostasis protein, is the first PLPBP family gene conferring resistance to heat stress during heading stage cloned by forward genetics in rice.

### The SNP variation in promoter increases transcript expression level and enhances heat tolerance at the heading stage

Several reports have shown that some grain yield‐related traits are influenced by gene expression differences which result from sequence variation in the promoter region in rice, such as the quantitative trait genes, *FZP*, *NOG1*, and *TGW2* (Bai *et al*., 2017; Huo *et al*., 2017; Ruan *et al*., 2020). In this study, we found that a T to C substitution at −325SNP (specific to HHT3) and −402SNP variations (constructs with A to G) in the promoter region of *HTH5* resulted in an increased level of expression, thus leading to an increase in PLP content under heat stress conditions. A heat stress test at the heading stage showed that an allele of *HTH5^HHT3^* could significantly increase grain yield compared to the control, indicating that it was a favourable gene for improved rice production in a high‐temperature environment.

### *HTH5* might contribute to heat tolerance by regulating ROS homeostasis under heat stress

*HTH5* belongs to the pyridoxal phosphate‐binding protein PLPBP (formerly called PROSC) family, which is predicted to encode pyridoxal phosphate homeostasis protein (PLPHP), and its orthologues (YggS, Ybl036c, and YlmE) are highly conserved and present in almost all kingdoms of life, including bacteria, plants, yeast, and mammals (Ito *et al*., 2013). In plant cells, the impairment of mitochondrial bioenergetics metabolism, often coupled with exaggerated reactive oxygen species (ROS) productio,is a fundamental oxidative damage mechanism in response to various types of abiotic stresses, such as drought, salt stress, and temperature extremes, (Apel and Hirt, 2004). Previous studies have reported that plants respond to abiotic stress by inducing ROS scavenging (Fang *et al*., 2015; Lee *et al*., 2012; Ning *et al*., 2010; Schmidt *et al*., 2013; Wu *et al*., 2012; Xiong *et al*., 2018). For example, overexpression of an *ERF* family transcription factor, *OsLG3*, can significantly improve drought tolerance in rice by inducing reactive oxygen species scavenging (Xiong *et al*., 2018). Overexpression of the NAC protein, *SNAC3*, significantly improves the heat tolerance of rice plants by modulating ROS homeostasis (Fang *et al*., 2015). Heat stress has been believed to be an environmental factor inducing ROS production, and it is the main production and targeting effect sites also have proven to be in mitochondrion (Belhadj Slimen *et al*., 2014). Heat‐induced oxidative damage changes ETC, ATP synthesis, uncoupling respiration, and structural characterization of enzymes, lipids and proteins. Because the heat stress response is unable to repair damaged proteins and block oxidative damage propagation, it is no wonder that heat stress is usually connected with significant decrease in cell viability. It has been reported that PLP (a pyridoxine derivative) is an efficient singlet oxygen quencher and a potential fungal antioxidant; it is also found that plays the role of redox quencher for excited cercosporin by forming the cercosporin radical anion under with electron paramagnetic resonance observing (Bilski *et al*., 2000).

In this study, *HTH5* encoded PLPHP was found to be localized in mitochondria, and we tested the hypothesis that *HTH5* may help regulate ROS homeostasis by PLP to alleviate the impairment of mitochondrial bioenergetics metabolism under conditions of heat stress. Consistent with the original hypothesis, pollen grains of Sasanishiki had higher signals from the general ROS sensor CM‐H_2_DCFDA under heat stress. To determine whether the lower ROS levels measured in R05‐12‐01 caused elevated pollen viability and seed‐setting rate, we detected transgene‐positive plant signals from a general ROS sensor CM‐H_2_DCFDA. Pollen grains of HTH5‐OE plants showed significantly reduced sensitivity to heat stress and lower ROS levels. In contrast, the pollen grains of the HTH5‐RNAi plants showed a significant increase in sensitivity to heat stress and elevated ROS levels. Furthermore, the measured results of H_2_O_2_ and MDA content showed that the accumulation in the panicles of HTH5‐OE plants was significantly lower than that in the wild‐type and HTH5‐RNAi plants (Figure 5b, c), indicating that the improved heat tolerance of HTH5‐OE plants was caused by quickening ROS scavenging and reducing levels of MDA, thereby lowering membrane lipid peroxidation. These results present that the function of *HTH5* in heat tolerance has a close connection with the regulation of antioxidant ability.

### A valuable heat tolerance gene resource for heat‐tolerant breeding

During crop anti‐abiotic stress breeding improvement, natural genetic resources from wild germplasms may be able to eliminate abiotic stress damage to domesticated modern crops through an environmentally friendly and economical way (Bruce, 2012). In this study, *HTH5* under the *japonica‐type* genetic background of Sasanishiki increased the seed‐setting rate by ~30% under the heat stress at the heading stage. More importantly, R05‐12‐01 and *HTH5* overexpression transgenic lines possessed stable heat tolerance not only in the controlled chamber but also in open field conditions, and did not affect other grain yield‐related traits (Figure S7; Table S1). Thus, the common wild rice, *O. rufipogon*, could offer an excellent natural genetic resource for the heat tolerance at the heading stage to increase heat‐induced spikelet sterility, and the discovery of novel heat tolerance genes could be of great potential for molecular breeding in rice to cope with hotter climates.

## Materials and methods

### Plant materials and development of NIL, R05‐12‐01

To investigate the genetic efficacy of *qHTH5* in different backgrounds, we used YJ10‐03‐01, a heat‐tolerant introgression line developed by HHT3 as a donor parent and an elite *indica‐type* cultivar Shuhui527 (*O. sativa indica*) as the recipient parent, to backcross with a heat‐sensitive *japonica‐type* cultivar Sasanishiki (*O. sativa japanica*) five times and selfing two times since 2012, to generate primary and fine mapping populations (Figure S1). In the winter of 2014, we planted a large BC_3_F_2_ population (3, 660 plants) at Sanya breeding station, Hainan Province, China, and screened five flanking simple sequence repeat (SSR) markers on chromosome 5 target segment (Figure 1a) and 621 SSR markers evenly distributed over the genome for forward and background selections according to the method of Cao *et al*. (2020). Among these BC_3_F_2_ plants, a homozygous *qHTH5* target introgression region, R05‐12‐01, possesses 99.8% genetic background similarity. To generate BC_3_F_3_, R05‐12‐01 was used as a NIL (Sasanishiki+*qHTH5*) for subsequent experiments. For validation and fine mapping of *qHTH5*, BC_4_F_2,_ BC_5_F_2,_ and BC_5_F_3_ populations were generated from the cross of R05‐12‐01 × Sasanishiki (Figure S1).

### Evaluation of heat tolerance and QTL analysis

The rice artificial chamber (Eshengtaihe Ctrl Tech, China) were used to heat treatment for each line at the heading stage for seven days. The high‐temperature treatment condition has been set at 38 ± 0.5°C from 8:30 am to 3:00 pm, and the low‐temperature treatment condition was set ~30 ± 0.5°C from 3:00 pm to 8:30 am with relative humidity of ~75 ± 5%. The light intensity was set at 40,000 lx, and the photoperiod range was set 13 h light/11 h dark. The atmospheric concentration of CO_2_ also was controlled at ~2000 ± 50 ppm, and exchanged fresh air five times per hour (Cao *et al*., 2020). After the treatment, the plants grown in pots were transferred to natural conditions for normal growth. In July 2020 at Nanchang breeding station, open field tests at the heading stage provided the daily average atmospheric temperatures of 32–35°C, air temperature exceeded 36°C after 9:00, and the maximum temperature reached ~39°C around noon in 2020. At maturity stage, the main panicles of plants were evaluated spikelet fertility. To analyse the pollen viability under heat stress, triphenyltetrazolium chloride (TTC) method was applied in testing the viability of the mature pollen under heat stress condition. TTC stock solution (2% by weight) was immediately diluted to working solution (0.5% weight) with distilled water before use (Shanghai Uteam Biotechnology Co., Ltd.). For the *in vitro* germination test, the liquid medium for rice pollen germination medium composed of 20% sucrose, 10% PEG, 3 mmol/L Ca (NO_3_)_2_, 10 mg VB_1,_ and 40 mg/L boric acid. After 6–8 min, the pollen tube emerged and increased at a rate of over 5.0 μm·min‐1 in 30 min. The incubation temperature for germination ranged from 28 to 32 °C. When the culture was complete, germination was observed under a microscope (Olympus BX51). For subsequent analysis, the phenotypic mean values were compared using the statistical software SPSS 17.0 (SPSS Inc., Chicago, IL, USA). QTL analysis was performed in QTL IciMapping 4.1 (Meng *et al*., 2015).

### Map‐based cloning of *HTH5*

In the summer of 2016, The BC_4_F_2_ population consisting of 368 individuals for validation mapping, was grown in the Nanchang Breeding Station at Jiangxi Academy of Agricultural Sciences (NBS/JAAS). In the summer of 2017, the larger BC_5_F_2_ population containing 7, 648 individuals was grown at the Nanchang breeding station. In the summer of 2018, all BC_5_F_3_ progenies used to heat tolerance at the heading stage were grown at the Nanchang breeding station.

To fine‐mapping *qHTH5*, recombinant individuals were identified in BC_5_F_2_ using five SSR markers and two InDel (insertion and deletion) markers, using the DNA polymorphism between Japonica rice Nipponbare genome sequence database (http://rgp.dna.affrc.go.jp/) and indica rice 9311 genome sequence database (http://rice.genomics.org.cn). To confirm the accuracy of phenotype, at least 10 plants of each homozygous recombinant progeny were used as biological replicates in BC_5_F_3_ to obtain the mean seed‐setting rate. To analyse candidate genes in *qHTH5*, R05‐12‐01 and Sasanishiki were grown in pots until the heading stage, and approximately 10 plants from each group were exposed to 38 °C in a phytotron. After the initiation of heat stress at the heading stage, panicle tissue RNA samples at different time points (1, 2, 3, 4, 5, 6, 7, 8, 9, 10, 11, 12, 13, 14, 15 and 16 h) were extracted for quantitative RT‐PCR at different time points. The genomic DNA sequence of candidate *HTH5* gene was obtained and a 1.5‐kb promoter fragment was sequenced and compared. The primer sequences used for genotyping PCR and DNA cloning are listed Table S6.

### Construction of plasmid vectors and genetic transformation for functional analysis

For complementation analysis, we amplified a ~ 5.1 kb DNA fragment from HHT3, which containing the 1.5 kb upstream of ATG, the 3.1 kb genomic region and a 0.5 kb downstream of *HTH5*. After digested with PmeI and SmaI, it was ligated into the binary vector pMDC83 (Curtis and Grossniklaus, 2003). For overexpression plasmids, we amplified the full‐length coding sequences from the cDNA of HHT3 and Sasanishiki, and cloned into binary vector pMDC32 after digestion with AscI and PacI (Curtis and Grossniklaus, 2003), respectively. To silence the gene by an RNAi plasmid, the unique hairpin sequence with ~370bp cDNA inverted repeats was amplified from Sasanishiki, and ligated into the pTCK303 vector after digestion with SacI and SpeI (Wang *et al*., 2004) for generating the forward insertion. Further dsRNAi fragments obtained by digestion with BamHI and KpnI, and cloned into the same vector for generating a reverse insertion.

We transferred all plasmids into rice genetic transformation materials with Agrobacterium tumefaciens strain *EHA105* (Hiei *et al*., 1994). All the corresponding fragments were amplified with KOD Plus DNA polymerase (Toyobo), then sequenced and confirmed by the MegaBACE 4500 DNA analysis system (Amersham Biosciences). Primer sequences for vector constructions are listed in Table S6.

### Expression pattern analysis

The RNA samples from different tissues of R05‐12‐01 and Sasanishiki were extracted and quantitative RT‐PCR using an RNAiso Plus Kit (Takara). We conducted each experiment with three biological samples, and each sample with five technical replicates. *OsActin1* was selected as the reference gene.

For construction of GUS plasmid, we amplified a ~1.5 kb DNA fragments containing the *HTH5* promoter from HHT3 and Sasanishiki, then digested with PmeI and AscI, and cloned into the pMDC162 vector (Curtis and Grossniklaus, 2003). T_2_ transgenic homozygous rice plants containing pHTH5^HHT3^::GUS vector were acquired for GUS histochemical staining. The primer sequences for PCR are listed in Table S6.

### Subcellular localization

For construction ofthe GFP plasmid, we amplified the ORF sequence of *HTH5* from the cDNA of HHT3 except a stop codon, digested with SpeI and AscI, and subcloned into the pMDC83 vector (Curtis and Grossniklaus, 2003). The p35S::GFP vector and p35S::HTH5‐GFP vectors were transformed into *E*. *coli* DH5α. The bacterial solutions were cultured and expanded *in vitro*, and p35S::GFP and p35S::HTH5‐GFP plasmids were co‐transformed with the mitochondrion marker CD3‐991 (Nelson *et al*., 2007) for transient gene expression analysis using Arabidopsis mesophyll protoplast (Yoo *et al*., 2007). After 8–10 h of incubation at 25 °C under low light, the Arabidopsis protoplasts were used for transient expression fluorescence signal observation. The confocal microscope (Olympus FV1000) was used to observe green and red fluorescence. The laser with wavelength of 488 nm was used to excite GFP, and excite CD3‐991 using a laser with wavelength of 543 nm. The GFP and CD3‐991 emission wavelengths ranges were confirmed at 500–550 nm and 565–615 nm, respectively. The PCR primer sequences are given in Table S6.

### Transient expression assay of promoter activity

For transient transformation in Arabidopsis protoplasts, all mutated pHTH5::GUS fusion constructs and the internal transformation control (CaMV35S::LUC plasmid) were obtained. The ratios of glucuronidase (GUS) to luciferase (Luc) activities of the sampled protoplasts were calculated by measuring the reaction product MU of the protoplasts using a microplate fluorescence reader of fluorescence FLx800 (BIO‐TEK Instruments). Each construct was assayed with three biological replicates, and each with five technical replicates. Cis‐element analysis was performed using PLACE (http://www. dna. affrc. go jp/PLACE).

### Promoter sequence analysis

According to the method described by Balding (2006), the Fisher’s exact test was used to perform an association test between sequence variations on the promoter of HTH5 and the seed‐setting rate. Linkage disequilibrium (LD) between two loci, A and B, was calculated using the method described by Lin *et al*. (2012). All SNP data for haplotype analysis were obtained from DNA sequence of *HTH5* in 181 rice accessions and amplified by PCR with the primers were listed in Table S6.

### Pyridoxic 5′‐phosphate( PLP) measurement

The PLP content was detected by the method described by Zhang *et al*. (2004) with a few modifications. A pollen grain sample (0.5 g) was collected and transferred into a 2‐ml centrifuge tube, and ruptured by electric drill grinding. The pollen grains were immersed in solution with 0.5 mL of 1 mol/L HClO_4_, then were centrifuged at 10,000 × g for 10 min at 4°C. The supernatant was filtered using a microporous filter paper (0.45μm). The supernatant was filtered and analysed using the high performance liquid chromatography (HPLC) (Waters 600) linked to an XPODS‐A5μm 120A column (H&E, 250 × 4.6 mm), and the chromatographic column temperature was held at 30 °C. To analyse the supernatant, mobile phase A was mixed as follows:1% (v/v) CH3CN‐25mmol/L, KH_2_PO_4_ (pH2.5). To clean chromatographic column, the mobile phase B was mixed as follows:10% (v/v) CH3CN‐25 mmol/L, KH_2_PO_4_‐2 mmol/L, NaCLO_4_ (pH2.5). The flow rate was set at 0.5 mL/min. The fluorescence detector adjusts the excitation and fluorescence wavelengths to 290 and 395 nm, respectively. The gradient elution procedure was programmed as follows: A (25 min) ‐ B (15 min) ‐ A (25 min).

### Physiological measurements

For CM‐H_2_DCFDA staining of pollen grains, rice anthers were collected from spikelets before flowering and preserved in a 5‐mL conical centrifuge tube. Pollens were obtained from anthers by squashing after heat stress. Pollen was immersed in pollen viability solution (PVS, 250 mM sucrose, 1.25 mM Ca(NO_3_)_2_, 0.19 mM boric acid, 1 mM KNO_3_), containing 10 µM CM‐H_2_DCFDA (stock solution: 500 µM CM‐H_2_DCFDA in DMSO; ThermoFisher). Pollen grains were stained for 15–20 min at 25°C and fixed on a microscope slide. Pollen grains were then imaged by confocal microscopy (Olympus FV1000). The laser settings for CM‐H_2_DCFDA were as follows: CM‐H_2_DCFDA was excited with a 488‐nm laser and fluorescence was collected at 495–504/517–529 nm. To verify the absence of autofluorescence, we took advantage of non‐stained samples for CM‐H_2_DCFDA imaging. Quantification of CM‐H_2_DCFDA fluorescence intensity in pollen grains was performed in Fiji (Schindelin *et al*., 2012). The average CM‐H_2_DCFDA fluorescence intensity within the perimeter of the pollen grain was used for the statistical analyses.

Pollen grains with treatment under normal and high temperature (28/38 °C) conditions for 24 h were subjected to quantitative determination of H_2_O_2_ at the heading stage. Pollen samples were immediately placed in liquid nitrogen for H_2_O_2_ determination using the Amplex Red Hydrogen/Peroxidase Assay Kit (Molecular Probes, Invitrogen). Three biological replicates were used per sample in this study.

The extent of lipid peroxidation was determined with the method described by Duan *et al*. (2012) with minor modifications, according to its the pollen grains malondialdehyde (MDA) content, and the pollen grain samples (approximately 0.50 g) were immersed in liquid nitrogen and homogenized with 10 mL of 10% trichloroacetic acid (TCA) solution, and then was centrifuged at 4°C for 10 min with 12,000 × g. The MDA content values from supernatants were measured at 450, 532, and 600 nm with 0.6% thiobarbituric acid (TBA) respectively, following the method of Dhindsa *et al*. (1981).

### Accession numbers

The sequence data for the study was obtained from RGAP database (http://rice. plantbiology.msu.edu) with the accession numbers of *LOC_Os05g05720*, *LOC_Os05g05730*, *LOC_Os05g05740*, *LOC_Os05g05750*, *LOC_Os05g05760*, *LOC_Os05g05770*, *LOC_Os05g05780*, and *OsActin*.

## Author contributions

Jianlin Wan and Linfeng Yuan designed the research, and Zhibin Cao, and Huiwu Tang performed most of experiments and analysed the data. Zhibin Cao Yaohui Cai, and Xiaofeng Wu developed the NILs and undertook the evaluation of heat tolerance under normal and controlled conditions; Jialiang Zhao, Bohong Zeng, Xiuying Tang, Xuejing Zhu, Huimin Wang, and Ming Lu performed part of the experiments; and Jianlin Wan, Linfeng Yuan and Zhibin Cao conceived of the experiments and wrote the manuscript.

## Conflict of interests

The authors have no conflict of interest to declare.

## Supporting information


**Figure S1** Construction of the mapping population.
**Figure S2** Progeny test of homozygous recombinants delimited *qHTH5* to a region about 26.5 kb flanked by markers RM1366 and InDel‐1.
**Figure S3** Expression analysis of candidate genes in NIL, R05‐12‐01.
**Figure S4** Multiple sequence alignment of *HTH5* orthologs from various eukaryotes.
**Figure S5** Phylogenetic and protein similarity(PT, %) analysis of *HTH5* orthologs in eukaryotes.
**Figure S6** Expression of *HTH5* in transgenic lines.
**Figure S7** *HTH5^HHT3^* overexpression improves rice grain yield under heat stress.
**Figure S8** Histochemical staining assay of GUS activity in *HTH5^HHT3^* promoter::GUS transgenic rice.
**Figure S9** Promoter activity analysis.
**Figure S10** Transient expression assays of Sas, HHT3, and the nine types of *HTH5* promoters (Hap1‐Hap9) under heat stress condition.

**Table S1** The phenotypic performances of Sasanishiki and R05‐12‐01 under high temperature stress in phytotron and normal temperature field trials conditions at the heading stage at Nanchang in 2016.
**Table S2** Relation of pollen vitality, Pollen germination rate on culture medium and mean seed‐setting rate per plant under high temperature stress at the heading stage.
**Table S3** Genetic analysis of the heat tolerance at heading stage of BC_4_F_3_ population.
**Table S4** Putative genes in the 26.5 kb *qHTH5* region.
**Table S5** The information of 181 rice cultivars for haplotype analysis of *HTH5*.
**Table S6** Primers used in this study.
