# Supplementary material for: Natural variation of HTH5 from wild rice, Oryza rufipogon Griff., is involved in conferring high‐temperature tolerance at the heading stage
Source: Plant Biotechnol J. 2022 May 25;20(8):1591–605. doi: 10.1111/pbi.13835 (PMC9342620; doi:10.1111/pbi.13835)
Supplement: Supplementary file 1 — Figure S1 Construction of the mapping population. Figure S2 Progeny test of homozygous recombinants delimited qHTH5 to a region about 26.5 kb flanked by markers RM1366 and InDel‐1. Figure S3 Expression analysis of candidate genes in NIL, R05‐12‐01. Figure S4 Multiple sequence alignment of HTH5 orthologs from various eukaryotes. Figure S5 Phylogenetic and protein similarity(PT, %) analysis of HTH5 orthologs in eukaryotes. Figure S6 Expression of HTH5 in transgenic lines. Figure S7 HTH5HHT3 overexpression improves rice grain yield under heat stress. Figure S8 Histochemical staining assay of GUS activity in HTH5HHT3 promoter::GUS transgenic rice. Figure S9 Promoter activity analysis. Figure S10 Transient expression assays of Sas, HHT3, and the nine types of HTH5 promoters (Hap1‐Hap9) under heat stress condition. [file PBI-20-1591-s002.pdf]

Natural variation of *HTH5* from wild rice, *Oryza rufipogon* Griff., is involved in conferring high-temperature tolerance at the heading stage

Zhibin Cao[1], Huiwu Tang[2], Yaohui Cai[1], Bohong Zeng[1], Jialiang Zhao[1], Xiuying Tang[1], Ming Lu[1], Huimin Wang[1], Xuejing Zhu[1], Xiaofeng Wu[1] , Linfeng Yuan[1*], Jianlin Wan[1*]

[1]Rice National Engineering Research Center (Nanchang), Jiangxi Research and Development Center of Super Rice, Jiangxi Academy of Agricultural Sciences, Nanchang 330200, China

[2]College of Agriculture and Biology, Zhongkai University of Agriculture and Engineering, Guangzhou 510550, China

*Correspondence authors:

Linfeng Yuan (e-mail:lfyuan2003@outlook.com, Tel:+86 079187090653)

Jianlin Wan (e-mail: ncwanjl66@126.com, Tel: +86 079187090780)

Supporting information Figure S1-S10

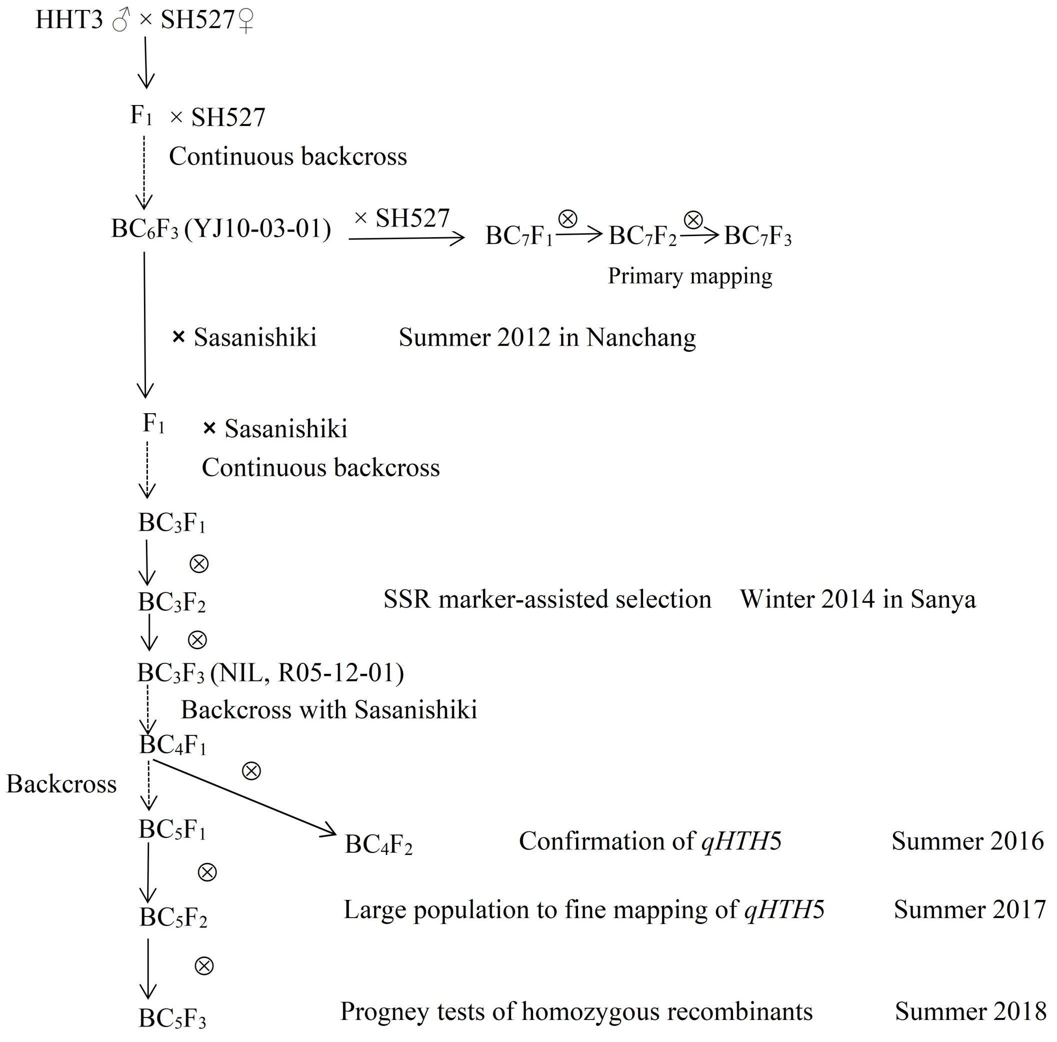

Figure S1 Construction of the mapping population.

YJ10-03-01 is a chromosome segment introgression line that contains a candidate QTL segment on chromosome 5 for *qHTH5*. R05-12-01 is a near-isogenic line containing the candidate QTL segment on chromosome 5 from HHT3, which was developed using YJ10-03-01 as a donor parent and the Japonica-type cultivar Sasanishiki as the recipient parent.

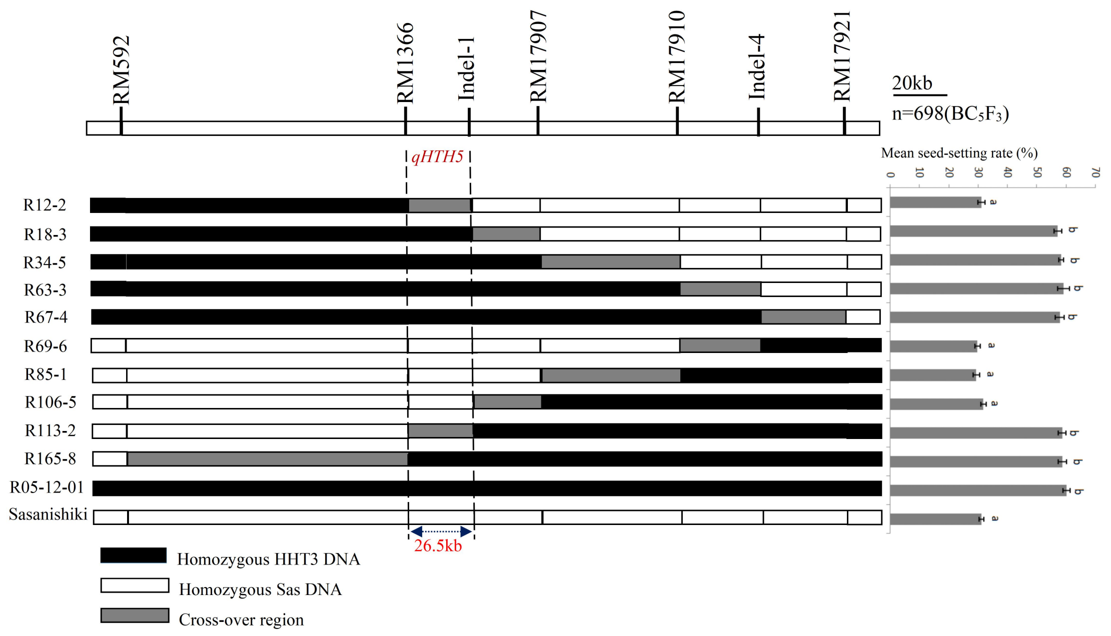

Figure S2 Progeny test of homozygous recombinants delimited *qHTH5* to a region of 26.5 kb flanked by markers RM1366 and InDel-1.

The mean spikelet fertility of the main panicles of each recombinants group is indicated in the bar diagram. Data represent means ± SD (n=10) Significance was determined using ANOVA, followed by comparisons of means using Tukey's test ($p < 0.05$). Same lowercase letters above the error bars denote non-significant difference between the means ($P > 0.05$, p-values were calculated using Student's *t*-test).

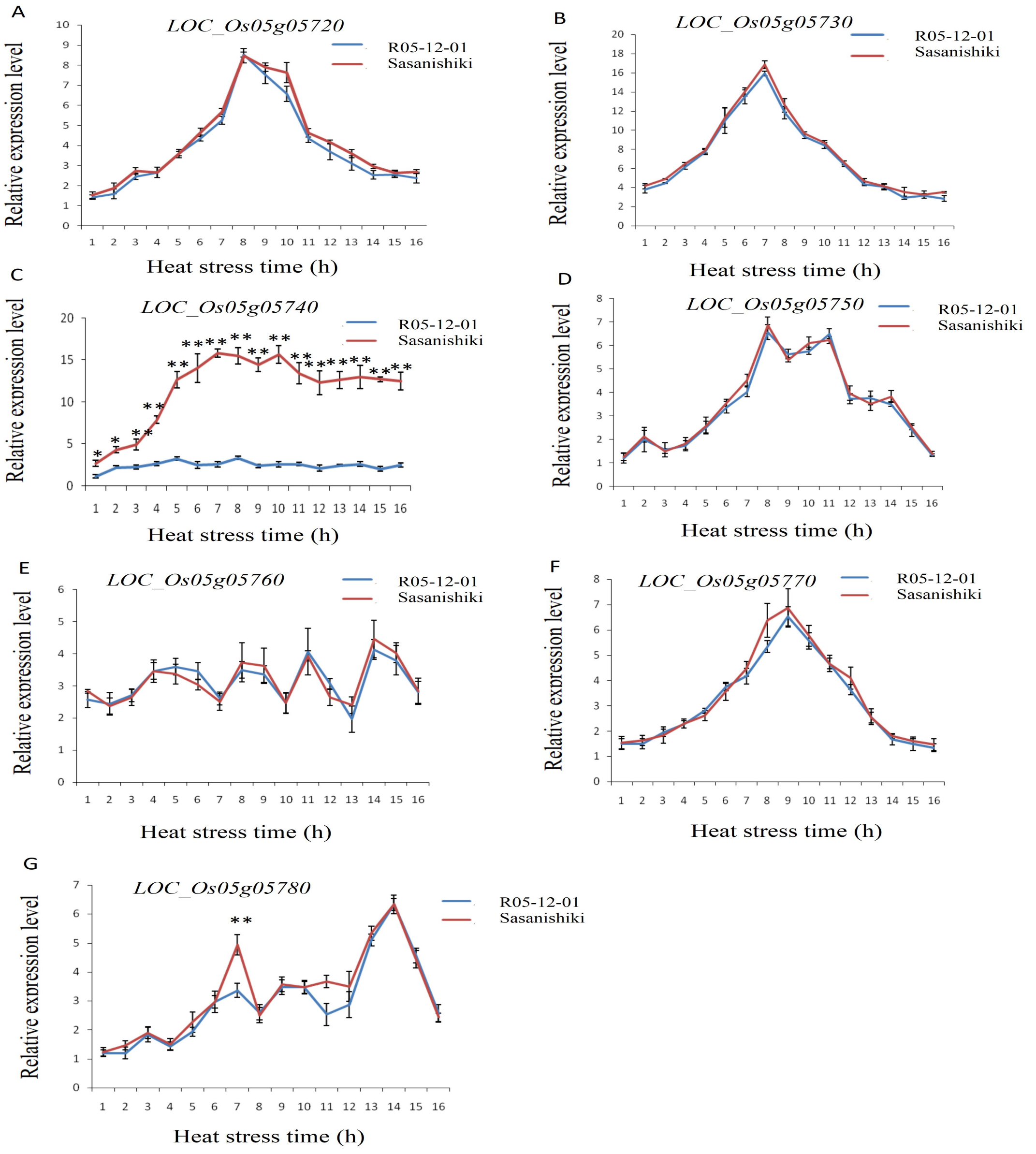

Figure S3 Expression analysis of candidate genes in NIL, R05-12-01.

(a-g) The panicle tissues exposing to 38 °C heat stress at heading stage sampled at different time points after the initiation of heading stage, namely, 1-16 h. Data represented by means ± SD (n=3), Student's *t*-test. ** indicate significant differences at the 0.01 level.

```
XP_020869798.1 Arabidopsis lyrata           --------------------MSAAAIDGV--AALRSVVQRVNQAAEKAGRVSDQIRVVAV   38
XP_010493541.1 Camelina sativa              MNDLENEEKPENEHVVESAMSSTAAIDGV--AALRSVFQRVNQAAEKAGRASDRVRVVAV   58
VVA21546.1     Prunus dulcis                -------------------MASSAAIDGVAAAALRSVMQRVQLAAQKSGRVAHEIRVVAV   41
TYI78069.1     Gossypium mustelinum         ------------------MAASAGAMDGLAASALRSVLQRVHQTAERCGRESQRIRVVAV   42
THU45716.1     Musa balbisiana              --------------------MSAPAVEGSAAAAFRSVLARVHQAAERSGRSPEQVRVVAV   40
XP_003568925.1 Brachypodium distachyon      --------------------MASAAAAEGAAAALRSVLSRAQQAAARSGRAPESVRVVAV   40
VAH14520.1     Triticum turgidum            --------------------MASVAAVEGAAAALRSVLSRAQQAAARAGRAPESVRVVAV   40
HTH5           Oryza sativa                 --------------------MASVAAAEGAAAALRSVLSRAQQAAARSGRAPESVRVVAV   40
NP_001130828.1 Zea mays                     --------------------MASVAAAEVAAAALRSVLSRAQQAAARAGRAPGSVRVVAV   40
XP_002440599.1 Sorghum bicolor               --------------------MASVAAAEGAAAALRSVLSRAQQAAARAGRAPGSVRVVAV   40
                                                                 :: *      :*:***. *.: :* :.**    :*****

XP_020869798.1 Arabidopsis lyrata           SKTKPVSLIRQVYDAGHRSFGENYVQEIIEKSPQLPDDIEWHFIGNLQSNKVKPLLSGVP   98
XP_010493541.1 Camelina sativa              SKTKPVSLIRQVYDAGHRSFGENYVQEVIEKAPQLPEDIEWHFIGNLQSNKVKPLLTGVP   118
VVA21546.1     Prunus dulcis                SKTKPVSVLRQVYDAGHRCFGENYVQELVEKAPQLPEDIEWHFIGNLQSNKVKPLLTGVP   101
TYI78069.1     Gossypium mustelinum         SKTKPVSVIRQVYDAGHRCFGENYVQEIVEKAPQLPDDIEWHFIGNLQSNKVKPLIAGVP   102
THU45716.1     Musa balbisiana              SKTKPASLLRQVYDAGHRCFGENYVQEIVEKARQLPVDIEWHFIGNLQRNKVKSLLAGVP   100
XP_003568925.1 Brachypodium distachyon      SKTKPVGVIRGVYDAGHRCFGENYVQELIDKAPQLPEDIEWHFIGNLQSNKAKALLAGVP   100
VAH14520.1     Triticum turgidum            SKTKPVGVIRGVYDAGHRCFGENYVQELIDKAPQLPEDIEWHFIGNLQSNKAKALLAGVP   100
HTH5           Oryza sativa                 SKTKPVGVIRGVYDAGHRCFGENYVQELIDKASQLPEDIEWHFIGNLQSNKARALLAGVP   100
NP_001130828.1 Zea mays                     SKTKPVPVIRGVYDAGHRCFGENYVQELIDKAPQLPEDIEWHFIGNLQSNKARALLAGVP   100
XP_002440599.1 Sorghum bicolor               SKTKPVPVIRGVYDAGHRCFGENYVQELIDKAPQLPEDIEWHFIGNLQSNKARALLAGVP   100
                                            *****. ::* *******.********:::*: *** *********** **.: *::***

XP_020869798.1 Arabidopsis lyrata           NLVMVESVDDEKIANMLDRVVGNIGRKPLKVLVQVNTSGEESKFGVEPSGCVGLAKHVKE   158
XP_010493541.1 Camelina sativa              NLVMVESVDDEKIANTLDRVVGNIGRKPLKVLVQVNTSGEESKFGVEPSGCVGLAKHVKE   178
VVA21546.1     Prunus dulcis                NLAMVESVDDKKIANRLDTVVASIGRKPLKVLLQVNTSGEESKSGVEPSGCVELAKHVSL   161
TYI78069.1     Gossypium mustelinum         NLAMVETVDDEKIANHLNRVVGSFERKPLKVLVQVNTSGEESKSGVEPSGCVELVKHVSL   162
THU45716.1     Musa balbisiana              NLHMIESVDDEKIANHVDRVVASLGRKPLKVLVQVNTSGEESKFGVDPSGCVNLVRHVKL   160
XP_003568925.1 Brachypodium distachyon      NLDMVESVDDEKIANRLDRVVADLGRKPLKVLVQVNTSGEESKFGVDPSGCVGLAKHVKL   160
VAH14520.1     Triticum turgidum            NLDMVESVDDEKIANRLDRVVADLGRKPLKVLVQVNTSGEESKFGVDPSGCVGLAKHVKL   160
HTH5           Oryza sativa                 NLDMVESVDDQKIANRLDRVVADLGRKPLKVLVQVNTSGEESKFGVDPSGCVELAKHVKL   160
NP_001130828.1 Zea mays                     NLDMVESVDDEKIANRLDRVVADLGRKPLKVLVQVNTSGEESKFGVDPSGCMELAKHVKL   160
XP_002440599.1 Sorghum bicolor               NLDMVESVDDEKIASRLDRVVADLGRKPLKVLVQVNTSGEESKFGVDPSGCVELAKHVKL   160
                                            ** *:*:***:***. :: **..: *******:********** **:****: *.:**.

XP_020869798.1 Arabidopsis lyrata           ACSNLEFSGLMTIGMADYTSTPENFKLLAKCRSEVCKELGIPEEQCELSMGMSGDFELAI   218
XP_010493541.1 Camelina sativa              ACSNLEFSGLMTIGMADYTSTPENFKLLAKCRSEVCKELGIPEEQCELSMGMSGDFELAI   238
VVA21546.1     Prunus dulcis                GCPNLEFCGLMTIGMLDYTSTPENFKTLANCRTEVCKALGIPEEQCELSMGMSADFELAI   221
TYI78069.1     Gossypium mustelinum         NCPNLQFCGLMTIGMPDYTSTPENFKTLANCRSEVCKALGIPEEQCELSMGMSSDFELAI   222
THU45716.1     Musa balbisiana              GCPNLIFSGLMTIGMLDYSSTPENFKTLSNCRAEVCKELEIPEEQCELSMGMSADFEQAI   220
XP_003568925.1 Brachypodium distachyon      SCPNLVFSGLMTIGMLDYSSTPENFKALSSCRKEVCDELGIPEEQCELSMGMSADFEQAI   220
VAH14520.1     Triticum turgidum            SCPNLVFSGLMTIGMLDYSSTPENFKALTSCRKEVCNELGIPEEQCELSMGMSADFEQAI   220
HTH5           Oryza sativa                 GCPNLVFSGLMTIGMLDYSSTPENFKALANCRKEVCKELGIPEEQCELSMGMSADFEQAI   220
NP_001130828.1 Zea mays                     NCPNLVFSGLMTIGMLDYSSTPENFKALANCREEVCKELGIPEEQCELSMGMSADFEQAI   220
XP_002440599.1 Sorghum bicolor               NCPNLVFSGLMTIGMLDYSSTPENFKTLANCREGVCKELGIPEEQCELSMGMSADFEQAI   220
                                             * ** *.******* **:******* *:.**  **. * *************.*** **

XP_020869798.1 Arabidopsis lyrata           ELGSTNVRIGSTIFGAREYPKK---   240
XP_010493541.1 Camelina sativa              ELGSTNVRIGSTIFGAREYPKKK--   261
VVA21546.1     Prunus dulcis                ELGSTNVRIGSTIFGAREYPKKLSN   246
TYI78069.1     Gossypium mustelinum         EMGSTNVRVGSTIFGAREYPKKN--   245
THU45716.1     Musa balbisiana              EMGSTNVRIGSTIFGVREYKEKDK-   244
XP_003568925.1 Brachypodium distachyon      EMGSTNVRVGSTIFGAREYPKKN--   243
VAH14520.1     Triticum turgidum            EMGSTNVRVGSTIFGAREYPKKN--   243
HTH5           Oryza sativa                 EMGSTNVRVGSTIFGAREYPKKN--   243
NP_001130828.1 Zea mays                     EMGSTNVRVGSTIFGAREYPKKN--   243
XP_002440599.1 Sorghum bicolor               EMGSTNVRVGSTIFGAREYPKKN--   243
                                            *:******:******.*** :*
```

Figure S4 Multiple sequence alignment of *HTH5* orthologs from various eukaryotes.

Full-length sequences of *HTH5* orthologues from various eukaryotes were used for multiple sequence alignments. Residues identical to HTH5 are marked with asterisks(*). Conservation between amino acids with strongly and weakly similar properties is indicated by the colon (:) and period (.), respectively. Multiple sequence alignments were performed using the ClustalW software. Species names and protein accession numbers are indicated on the left.

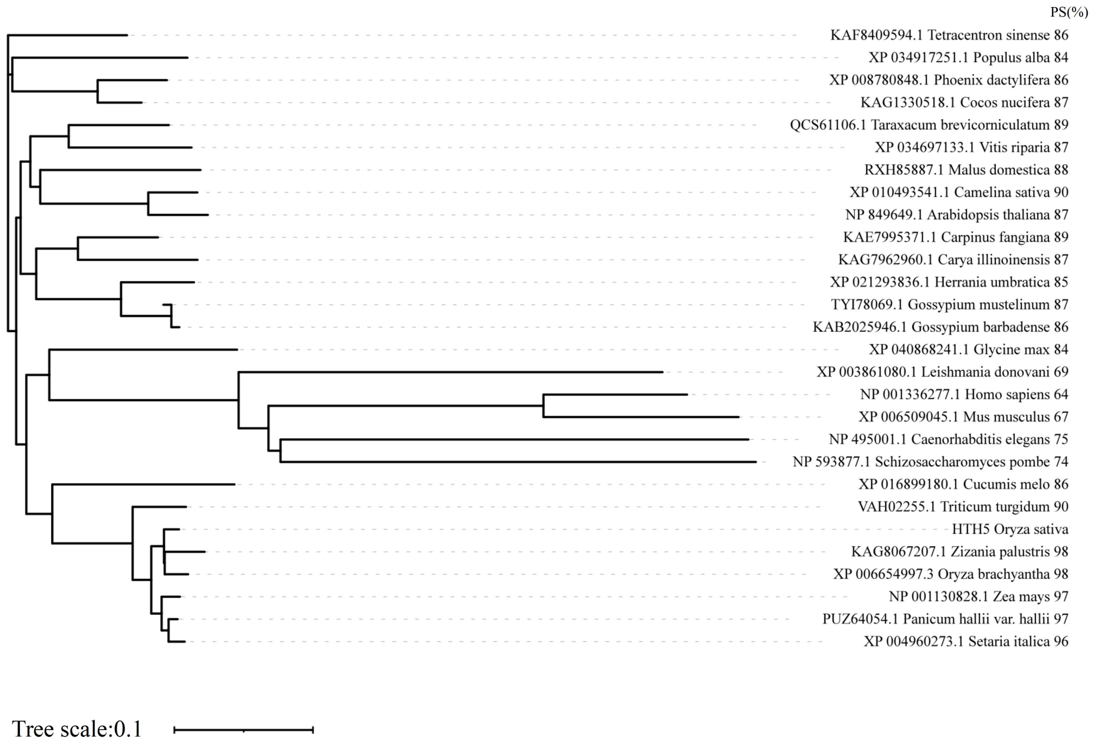

Figure S5 Phylogenetic and protein similarity (PT, %) analysis of *HTH5* orthologs in eukaryotes.

Full-length sequences of *HTH5* orthologues from various eukaryotes were obteined using by BLAST. Multiple sequence alignments of the *HTH5* sequences were performed using Clustaw software. The neighbour-joining tree was constructed using MEGA 6.0 with default parameters1, 000 bootstrap replicates. The cut-off value for the condensed tree was 50. Numbers indicate bootstrap support based on 1,000 replicates and branch length indicates substitutions per site. Protein accession numbers, species names, and protein similarities between *HTH5* and each orthologue are indicated on the right.

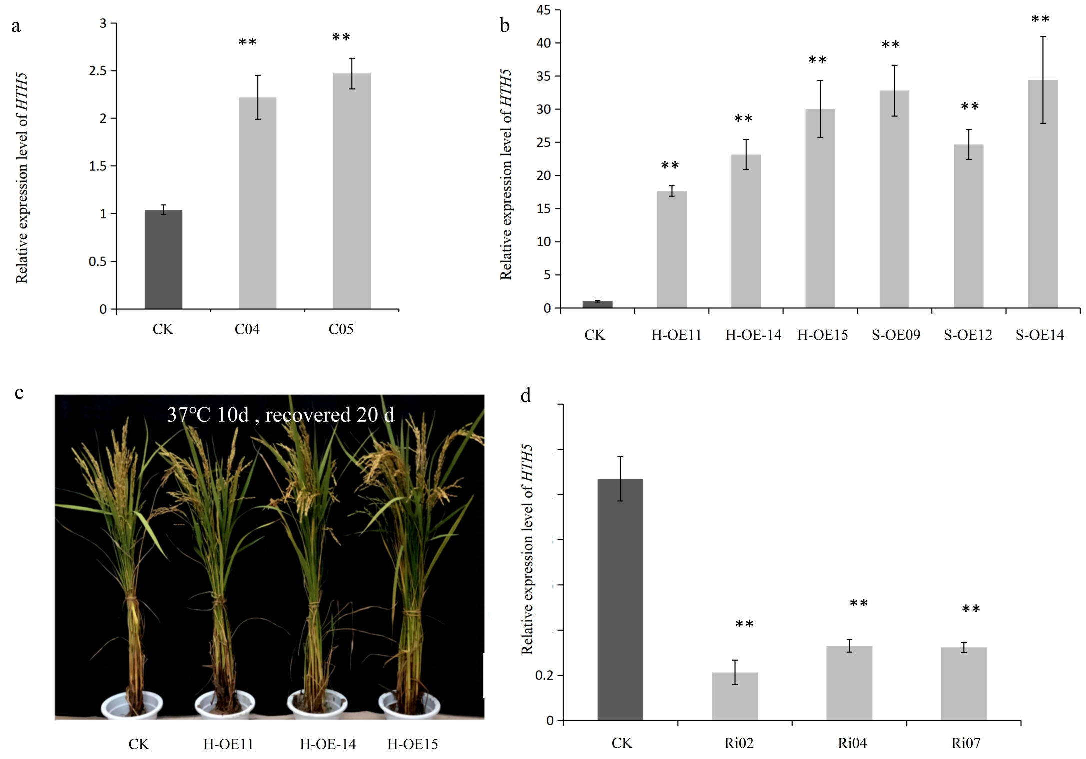

Figure S6 Expression of *HTH5* in transgenic lines.

(a) *HTH5*^{HHT3} complementation lines C04 and C05. CK, transgene-negative control.

(b) *HTH5*^{HHT3} overexpressing lines H-OE11, H-OE14, and H-OE15; *HTH5*^{Sas} overexpressing lines S-OE09, S-OE12, and S-OE14. CK, transgene-negative control.

(c) Differential heat tolerance of negative(CK) and positive(H-OE) transgenic lines under heat treatment at the heading stage. Scale bar, 10cm.

(d) *HTH5* RNAi lines in NIL(Sasanishiki) background.CK, transgene-negative control; Ri02, Ri04 and Ri05, transgene-positive lines.Values are represent as mean ±SD (n=3). Significant differnces were determined by Student's *t*-test) (**p < 0.01, a,b, and d).

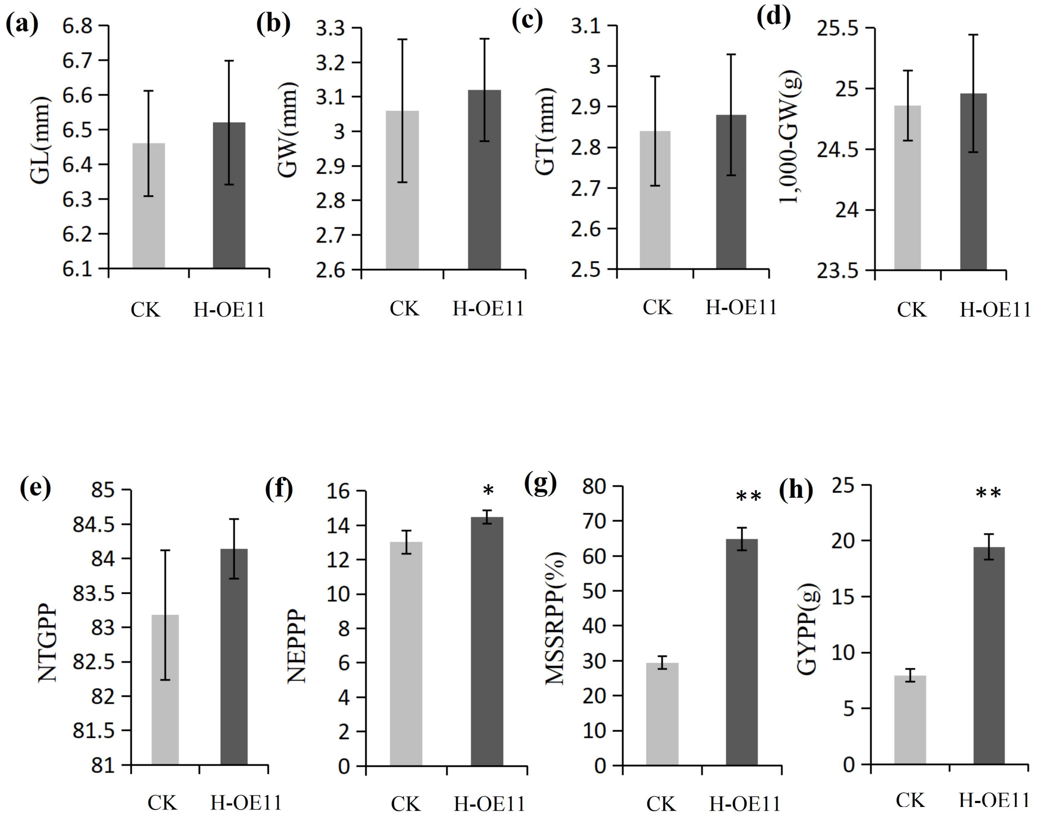

Figure S7 *HTH5*^{HHT3} overexpression improves rice grain yield under heat stress.

(a-h) Comparison of yield-related agronomic traits of the *HTH5*^{HHT3} overexpression lines and controls under heat treatment at the heading stage. CK, transgene-negative control; H-OE11, transgene-positive line. GL,Grain length; GW, Grain Width; GT, Grain thickness; NEPPP, No. of effective panicles per plant; NTGPP, No. of total grains per panicle; MSSRPP, Mean seed-setting rate per plant(%); 1000-GW, 1000-grain weight; GYPP, Grain yield per plant. Data represent mean ± SD(n=5). Significant differences were determined by Student's *t*-test. *, and ** indicate significant differences at the 0.05 and 0.01 level, respectively.

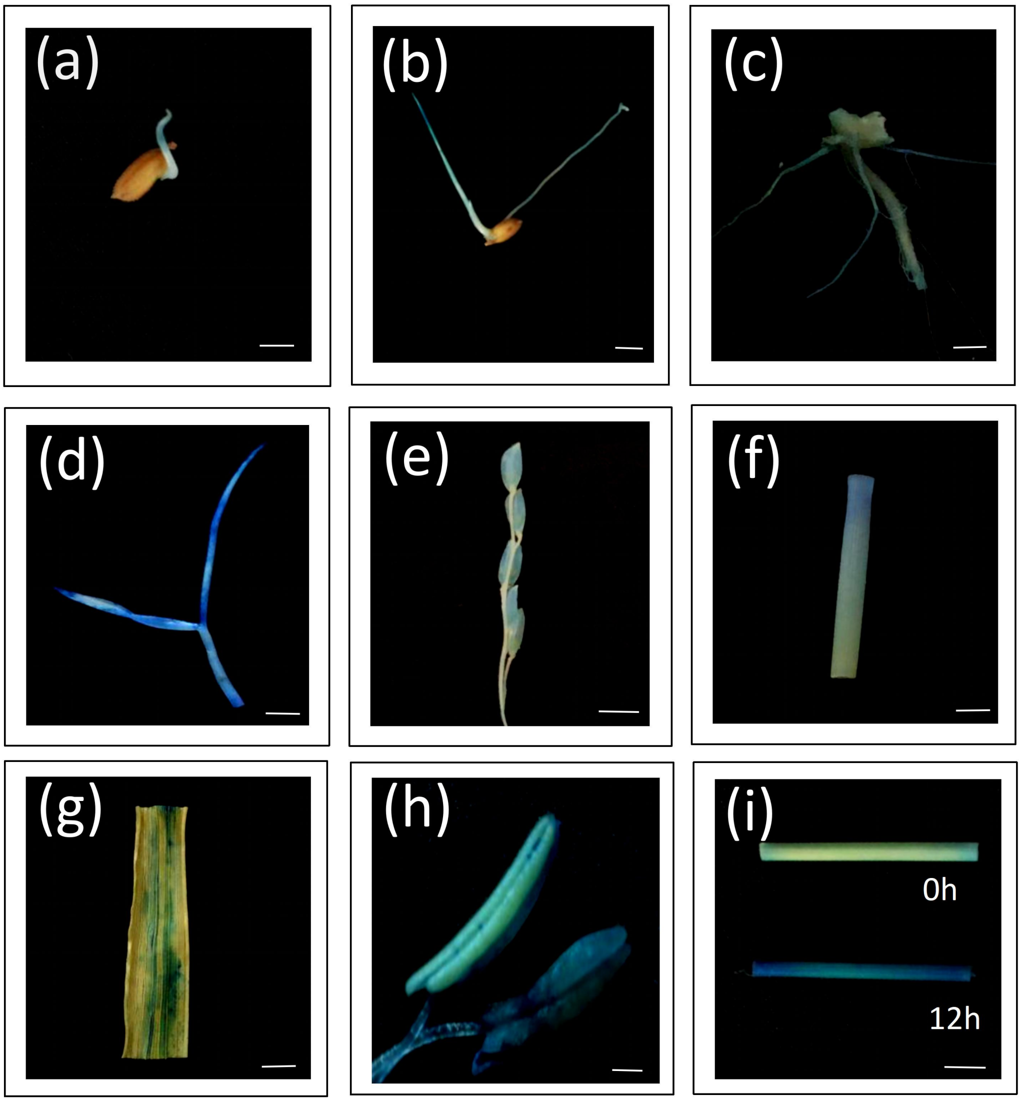

Figure S8. Histochemical staining assay of GUS activity in *HTH5*^{HHT3} promoter::GUS transgenic rice.

(a) Bud at 3 d after germination. (b) Bud at 5 d after germination. (c) Roots at 14 d after germination. (d) Young leaves at 14 d after germination. (e) Spikelet hulls at the booting stage.

(f) Elongation internode at the heading stage. (g) Flag leaf at the heading stage. (h) Mature anthers before dehiscence. (i) GUS expression increased in leaf sheath after 12 h treatment under 38°C. This analysis was repeated several times, yielding similar results. (a-g, i) bars, 5 mm; (h) bar, 500μm.

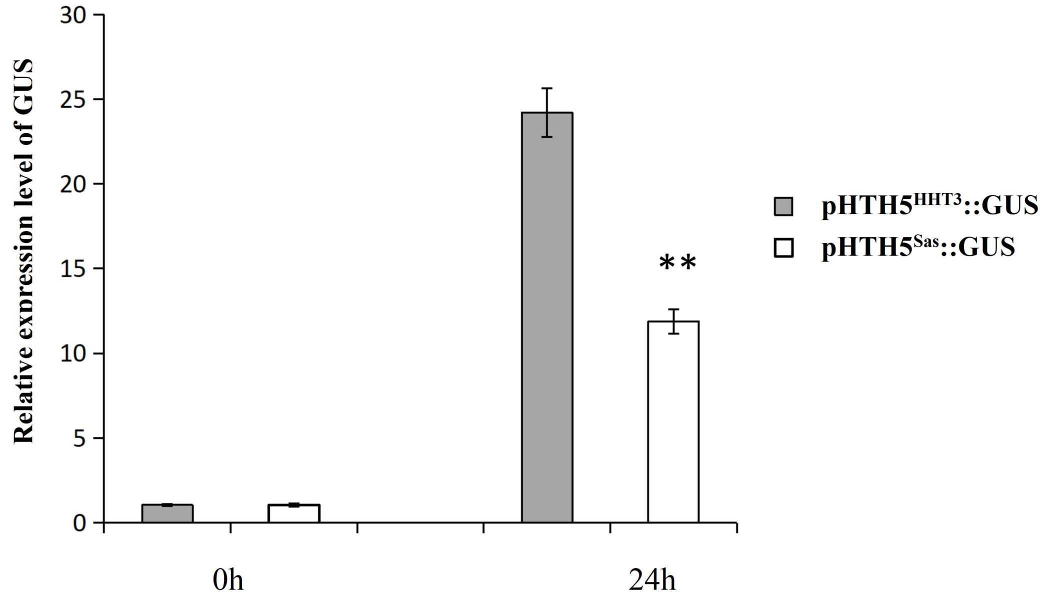

Figure S9 Promoter activity analysis.

Transgenic lines with single copy of pHTH5^{HHT3}::GUS or pHTH5^{Sas}::GUS were isolated and subjected to heat stress (24h/38 °C). At least three RNA samples were prepared and each experiment was performed with five technical repeats. Data represent mean ± SD (n=3). **P < 0.01. Student's *t*-test.

(a)

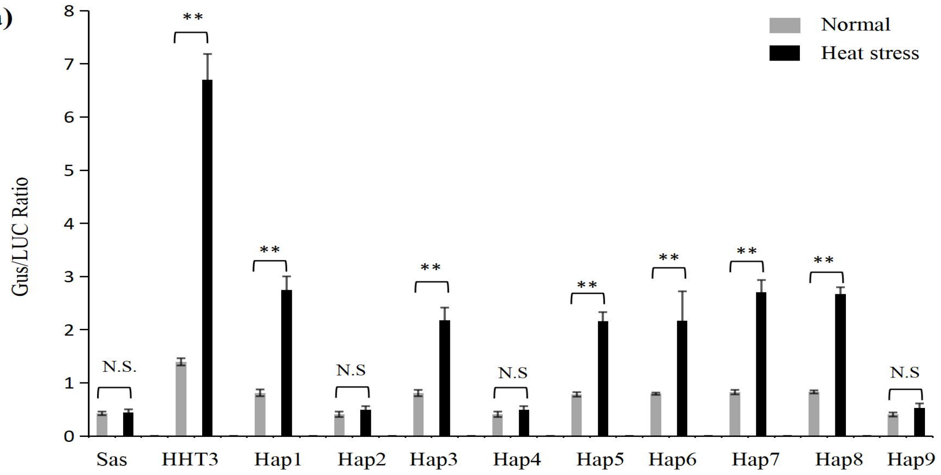

(b)

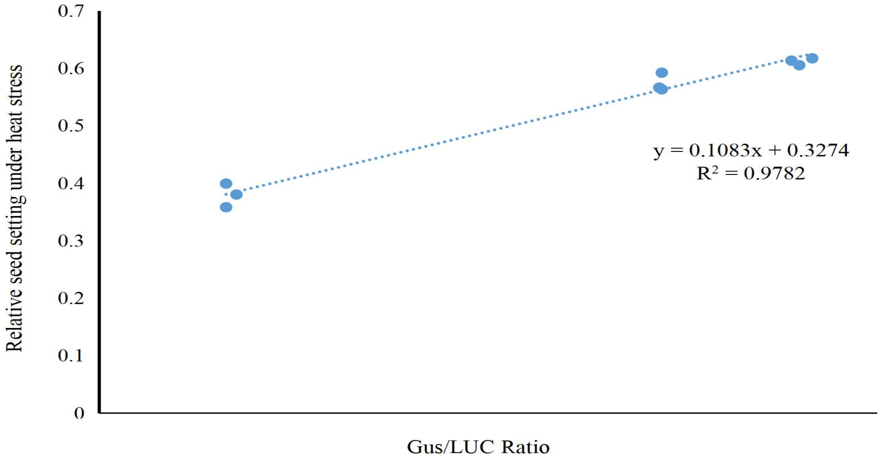

Figure S10 Transient expression assays of Sas, HHT3 and the nine types of *HTH5* promoters (Hap1-Hap9) under heat stress condition.

(a) Transient expression assays of Sas, HHT3 and the nine types of promoters (Hap1-Hap9) in *Arabidopsis* protoplasts harbouring transient expression constructs under normal condition (8 h/28 ℃) and heat stress treatment (8 h/38 ℃). Data represent mean ± SD (n=10). N.S. and ** denote non-significant differences and significant differences between means ($p < 0.01$, student's *t*-test), respectively. Internal control: 35S::LUC construct.

(b) Linear regression between relative seed-setting rate and relative expression activities driven by *HTH5* promoters of Hap1-Hap9 under heat stress.
